# Discovery and Optimization of Tau Targeted Protein Degraders Enabled by Patient Induced Pluripotent Stem Cells-Derived Neuronal Models of Tauopathy

**DOI:** 10.3389/fncel.2022.801179

**Published:** 2022-03-03

**Authors:** M. Catarina Silva, Ghata Nandi, Katherine A. Donovan, Quan Cai, Bethany C. Berry, Radoslaw P. Nowak, Eric S. Fischer, Nathanael S. Gray, Fleur M. Ferguson, Stephen J. Haggarty

**Affiliations:** ^1^Chemical Neurobiology Laboratory, Department of Neurology and Psychiatry, Center for Genomic Medicine, Massachusetts General Hospital, Boston, MA, United States; ^2^Department of Neurology, Harvard Medical School, Boston, MA, United States; ^3^Department of Cancer Biology, Dana-Farber Cancer Institute, Boston, MA, United States; ^4^Department of Biological Chemistry and Molecular Pharmacology, Harvard Medical School, Boston, MA, United States

**Keywords:** tau, structure-activity relationships, targeted protein degradation, PROTAC, human stem cells, human neuronal models, frontotemporal dementia

## Abstract

Accumulation of misfolded, aggregating proteins concurrent with disease onset and progression is a hallmark of neurodegenerative proteinopathies. An important class of these are tauopathies, such as frontotemporal dementia (FTD) and Alzheimer’s disease (AD), associated with accumulation of aberrant forms of tau protein in the brain. Pathological tau undergoes abnormal post-translational modifications, misfolding, oligomerization and changes in solubility, cellular redistribution, and spreading. Development and testing of experimental therapeutics that target these pathological tau conformers requires use of cellular models that recapitulate neuronal endogenous, non-heterologous tau expression under genomic and physiological contexts relevant to disease. In this study, we employed FTD-patient induced pluripotent stem cells (iPSC)-derived neurons, expressing a tau variant or mutation, as primary models for driving a medicinal chemistry campaign around tau targeting degrader series. Our screening goal was to establish structure-activity relationships (SAR) for the different chemical series to identify the molecular composition that most efficiently led to tau degradation in human FTD *ex vivo* neurons. We describe the identification of the lead compound QC-01-175 and follow-up optimization strategies for this molecule. We present three final lead molecules with tau degradation activity in mutant neurons, which establishes potential disease relevance and will drive future studies on specificity and pharmacological properties.

## Introduction

Tauopathies, such as frontotemporal dementia (FTD) and Alzheimer’s disease (AD), are neurodegenerative diseases characterized by the aberrant accumulation of misfolded, insoluble, and hyper-phosphorylated tau (P-tau) protein within neurons and glia of specific brain regions (Kosik et al., 1989; Goedert, 2004; Morris et al., 2011; Cruts and Van Broeckhoven, 2015; Ghetti et al., 2015; Neumann et al., 2015; Olney et al., 2017). Tauopathies can be sporadic or inherited when caused by mutations in the *MAPT* gene encoding the microtubule-associated protein tau (Rohrer et al., 2009; Benussi et al., 2015), but the molecular mechanisms leading to neuronal toxicity and death, and therefore potential therapeutic targets, are still not fully understood (Gotz et al., 2013; Panza et al., 2016; Congdon and Sigurdsson, 2018; Medina, 2018). Consequently, and despite a high prevalence in individuals under 60 years of age and escalating socioeconomic burden, there are currently no effective disease-modifying therapies, and few experimental drugs focused on tau have reached clinical trials (Dai et al., 2017; DeVos et al., 2017; Jadhav et al., 2019; Sandusky-Beltran and Sigurdsson, 2020).

**FIGURE 1:**
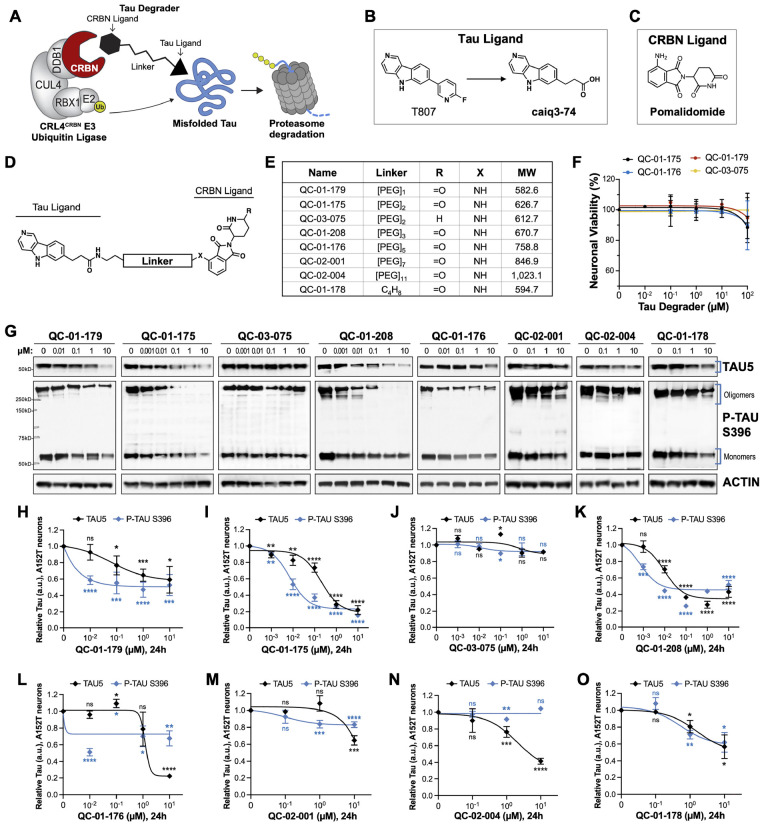
Design strategy for CRBN-recruiting degraders and concentration effect on tau and P-tau of human FTD neurons. **(A)** Working model for hetero-bifunctional tau degraders designed to preferentially recognize pathological forms of tau (misfolded protein) and simultaneously engage with CRBN (CRL4*^CRBN^* E3 ubiquitin ligase complex). Formation of the ternary complex is expected to enhance tau ubiquitination and degradation by the proteasome. **(B–D)** Degraders were synthesized based on the T807 core scaffold for tau recognition **(B)**, a thalidomide analog as an E3 ligand (pomalidomide, **C**) for CRBN engagement, and a variable linker length and composition to maximize target clearance efficiency **(D)**. **(E)** Summary of the chemical properties of CRBN-recruiting degrader molecules of the QC-Series. **(F)** Neuronal viability of tau-A152T neurons at 6 weeks of differentiation treated with representative degraders of the series for 24 h. Viability is shown relative to vehicle-treated neurons (100%) and each data point represents the mean% viability ± SD, *n* = 3. **(G–O)** Concentration effect of degraders on tau protein of A152T neurons (6-week differentiated) by analysis of total tau (TAU5) and P-tau S396 levels upon treatment for 24 h. Representative western blots are shown **(G)** and brackets indicate bands quantified for TAU5 and P-tau S396 densitometry **(H–O)**. Data points represent mean densitometry normalized to actin and relative to vehicle ± SEM (*n* ≥ 3). Data points for QC-01-175 **(I)** and QC-03-075 **(J)** include values from new biological replicates averaged with analysis previously published (Silva et al., 2019). The 0 μM degrader data points correspond to vehicle alone (DMSO) control treatment. Statistics: 2-tailed unpaired Student’s *t*-test for each concentration relative to vehicle; *^ns^P* > 0.05, *P < 0.05, **P < 0.01, ***P < 0.001, ****P < 0.0001.

Drug discovery against misfolded disease-associated proteins frequently utilizes heterologous model systems with recombinant protein overexpression and/or simplified cellular systems that do not accurately replicate key aspects of disease biology. By implementing FTD patient-specific neuronal models (Iovino et al., 2015; Silva et al., 2016, 2020; Wray, 2017; Jiang et al., 2018; Nakamura et al., 2019; Silva and Haggarty, 2020), we previously reported the first, non-peptidic, tau-selective degrader QC-01-175, developed for target validation and to aid phenotypic characterization of the consequences of targeted degradation in a tauopathy disease context (Silva et al., 2019). This approach focuses on targeting specific forms of tau in mutant neurons for clearance, halting disease-related molecular and cellular events in patient-derived *ex vivo* neurons, as a promising therapeutic strategy for reducing neurodegeneration. Targeted protein degraders, or PROTACs (PROteolysis TArgeting Chimeras), are bivalent molecules composed of a small molecule binder for the protein of interest (POI), i.e., tau, linked *via* a short chain to an E3-ligase (e.g., cereblon or CRBN) recruiting molecule such as an immunomodulatory drug (IMiD) (Figures 1A–C; Xie et al., 2014; Lai and Crews, 2017; Cromm et al., 2018; Gechijian et al., 2018). The degrader molecule recruits the POI and the E3-ligase simultaneously to form a ternary complex that promotes ubiquitination and proteasomal degradation of the POI (Figure 1A; Chamberlain et al., 2014; Fischer et al., 2014; Kronke et al., 2014; Lu et al., 2014; Metzger et al., 2014; Collins et al., 2017). We harnessed this strategy to transform one of the most experimentally and clinically advanced tau PET (positron emission tomography) imaging tracers, T807 or AV-1451 (flortaucipir) (Holt et al., 2016; Johnson et al., 2016; Scholl et al., 2016; Smith et al., 2016; Schonhaut et al., 2017; Jones et al., 2018; Murugan et al., 2018) into a CRBN-recruiting tau degrader (Chien et al., 2013; Xia et al., 2013; Lowe et al., 2016). Our first tau degrader was capable of clearing tau most effectively in mutant tau *ex vivo* patient neurons, suggesting a conformation-dependent selectivity toward misfolded tau, and had negligible effects on tau levels in control healthy neurons (Silva et al., 2019).

Here, we focus solely on structure-activity relationships (SAR) for different chemical series and their effect on tau of FTD iPSC-derived neurons to identify degraders that promote tau and P-tau reduction in a disease context. We first describe the SAR for the tau degrader series that culminated in the identification of the first-generation, lead compound QC-01-175. We then explore alternative chemical compositions and present evidence that CRBN-targeting degraders are more effective in promoting tau degradation in human neurons relative to alternative E3-ligases, such as the Von Hippel-Lindau (VHL). Finally, we introduce optimized derivatives of the lead molecule with improved tau reduction in FTD neuronal models, including degradation of insoluble tau and prolonged effect on tau levels. All studies were conducted in FTD-derived neurons expressing tau-A152T or tau-P301L to increase the potential for disease-relevance of the degraders selected through SAR.

## Materials and Methods

### Cereblon and Von Hippel-Lindau Cellular Engagement Assay

The E3 ligase *in vitro* binding assays were adapted from Nowak et al. (2018). Briefly, a day before compound treatment, cells stably expressing BRD4_*BD*2_-GFP with mCherry reporter were seeded at 30–50% confluency in 384-well plates (3764, Corning) with 50 μL FluoroBrite DMEM media (Gibco, A18967) containing 10% FBS per well. Compounds and either 250 nM AT-1 (Gadd et al., 2017) for the VHL engagement assay or 100 nM dBET6 (Winter et al., 2017) for CRBN engagement assay, were dispensed using D300e Digital Dispenser (HP) normalized to 0.5% DMSO and incubated with cells for 5 h. The assay plate was imaged immediately using Acumen eX3/HCl (TTPLabtech) High Content Imager with 488 and 561 nm lasers in 2 × 1 μm grid per well format. The GFP/RFP ratio of the resulting images was analyzed using a CellProfiler pipeline (Carpenter et al., 2006). The GFP/mCherry ratio was normalized to DMSO and fitted in GraphPad Prism 7 using variable slope equation.

### Human Neural Progenitor Cell Lines

Work with human iPSC and derived neural progenitor cell (NPC) lines was performed under the Massachusetts General Hospital/Mass General Brigham-approved IRB Protocol #2010P001611/MGH. Reprogramming of patient dermal fibroblasts into iPSCs by non-integrating methods and subsequent conversion into cortical-enriched neural progenitor cells were previously described (Silva et al., 2016; Seo et al., 2017). The cell lines employed in this work were as follows: NPC line FTD19-L5-RC6 (Silva et al., 2016) was derived from a male individual in his 60 s with Progressive Supranuclear Palsy (PSP) carrying the risk variant tau-A152T (c.1407G > A, rs143624519); NPC line MGH2046-RC1 (Seo et al., 2017; Silva et al., 2019) was derived from a female individual in her 50 s with FTD carrying the autosomal dominant mutation tau-P301L (c.C1907T, rs63751273); NPC Control-1 or 8330-8-RC1 (Sheridan et al., 2011; Silva et al., 2016) was derived from an unaffected male individual in his 60 s [tau wild type (WT)]; and NPC Control-2 or MGH2069-RC1 (Seo et al., 2017; Silva et al., 2019) was derived from an unaffected female individual in her 40 s (tau-WT).

### Neural Progenitor Cell Culture, Differentiation and Compound Treatment

**FIGURE 2:**
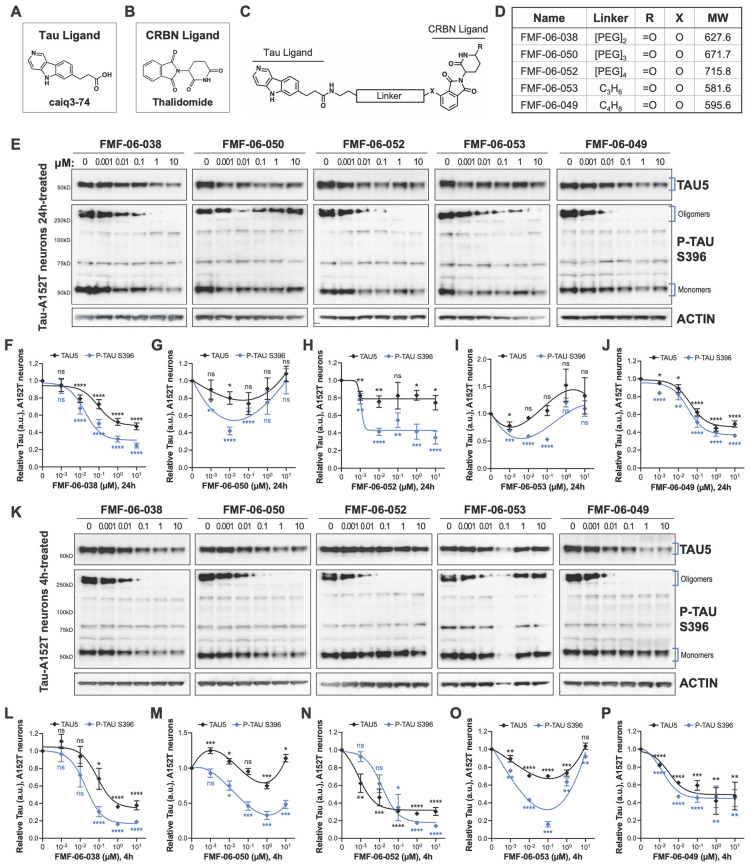
Characterization of second-generation CRBN-recruiting degraders (FMF-06-series) focusing on optimization of linker attachment chemistry. **(A–C)** The FMF-06-series was synthesized based on the T807 core scaffold for tau recognition **(A)**, thalidomide as the E3 ligand for CRBN engagement **(B)**, and a variable linker length and attachment composition **(C)**. **(D)** Summary of chemical properties of the FMF-06-Series. **(E–J)** Degraders’ concentration effect on tau protein of A152T neurons (6-week differentiated) with analysis of total tau (TAU5) and P-tau S396 levels upon treatment for 24 h. Representative western blots are shown **(E)** and brackets indicate bands quantified for TAU5 and P-tau S396 densitometry **(F–J)**. Data points represent mean densitometry normalized to actin and relative to vehicle ± SEM (*n* = 3). **(K–P)** Degraders’ concentration effect on tau protein levels of A152T neurons (6-week differentiated) after a 4 h treatment. Representative western blots are shown **(K)** for immunoprobing with TAU5, P-tau S396 and actin. Data points **(L–P)** represent mean densitometry normalized to actin and relative to vehicle ± SEM (*n* = 3). The 0 μM degrader data points correspond to vehicle alone (DMSO) control treatment. Statistics: 2-tailed unpaired Student’s *t*-test for each concentration relative to vehicle; *^ns^P* > 0.05, *P < 0.05, **P < 0.01, ***P < 0.001, ****P < 0.0001.

**FIGURE 3:**
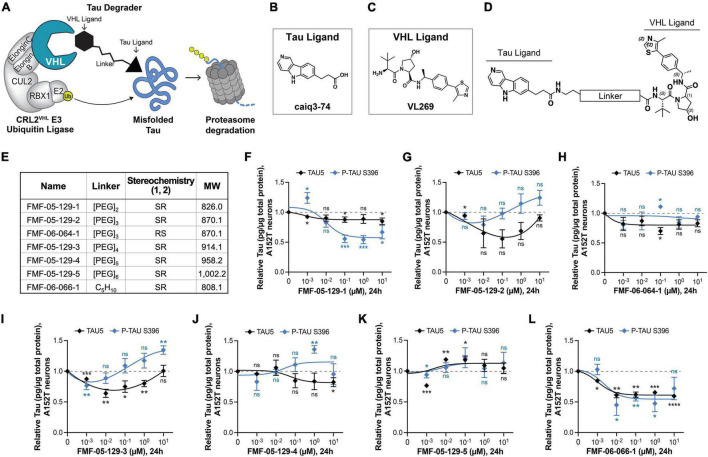
Design, proposed model, and testing of VHL-targeting tau degraders. **(A)** Hypothesis model for hetero-bifunctional tau degraders designed to simultaneously bind pathological misfolded tau and the substrate recognition component VHL within the CRL2*^VHL^* E3-ubiquitin ligase complex, to promote tau ubiquitination and proteasomal degradation. **(B–D)** The VHL series incorporates the T807 core scaffold for tau recognition **(B)**, the VHL ligand VL269 **(C)** and a variable linker length and composition **(D)**. **(E)** Summary of chemical properties for VHL-recruiting degrader molecules. **(F–L)** Concentration effect of VHL degraders on total tau (TAU5) and P-tau S396 levels in A152T neurons (6-week differentiated) after 24 h of treatment, measured by ELISA. Data points represent mean tau levels (μg of tau normalized to total protein in the lysate) relative to vehicle samples ± SEM (*n* = 3). The 0 μM degrader data points correspond to vehicle alone (DMSO) control treatment. Statistics: 2-tailed unpaired Student’s *t*-test for each concentration relative to vehicle; *^ns^P* > 0.05, *P < 0.05, **P < 0.01, ***P < 0.001, ****P < 0.0001.

**FIGURE 4:**
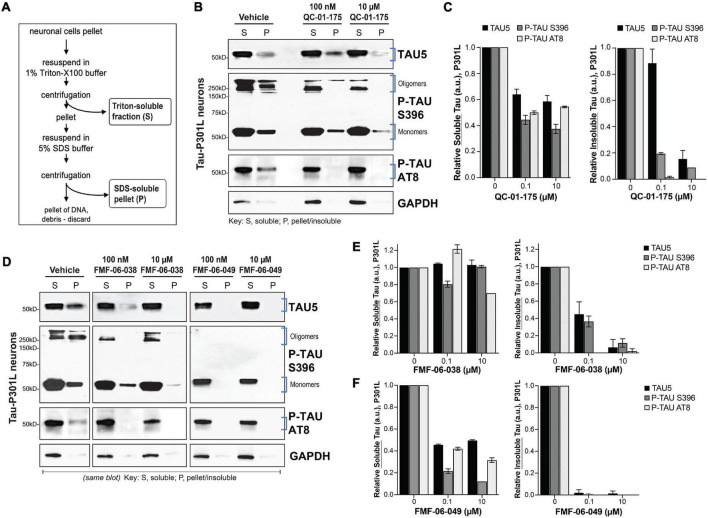
Lead tau degraders have a more pronounced effect on insoluble tau of FTD neurons. **(A)** Summary of the assay for protein fractionation, based on Triton-X (S, soluble) and SDS (P, pellet) detergents protein differential solubility. **(B,C)** Western blot and densitometry quantification of total tau (TAU5), P-tau S396 and P-tau S202/T205 (AT8) in the soluble (S) and insoluble-pellet (P) lysate fractions of P301L neurons (6-weeks differentiated) treated with QC-01-175 for 24 h at 100 nM or 10 μM. Brackets **(B)** indicate the bands corresponding to densitometry quantification in **(C)**. Graph bars represent mean densitometry ± SEM for soluble (*left*) and insoluble (*right*) tau levels relative to vehicle samples. *n* = 3 biological replicates. **(D–F)** Western blot analysis and densitometry quantification of total tau, P-tau S396 and P-tau S202/T205 (AT8) in the soluble (S) and insoluble-pellet (P) lysate fractions of P301L neurons treated with FMF-06-038 and FMF-06-049 for 24 h at 100 nM or 10 μM. Samples were run on the same blot **(D)**, and the image was cropped only for the purpose of this figure to exclude samples not included in the study. Brackets indicate protein bands in the densitometry analysis. **(E,F)** Graph bars represent mean densitometry ± SD for soluble (*left*) and insoluble (*right*) tau levels relative to vehicle samples. 0 μM data points correspond to vehicle alone (DMSO) control treatment. *n* = 2 biological replicates.

**FIGURE 5:**
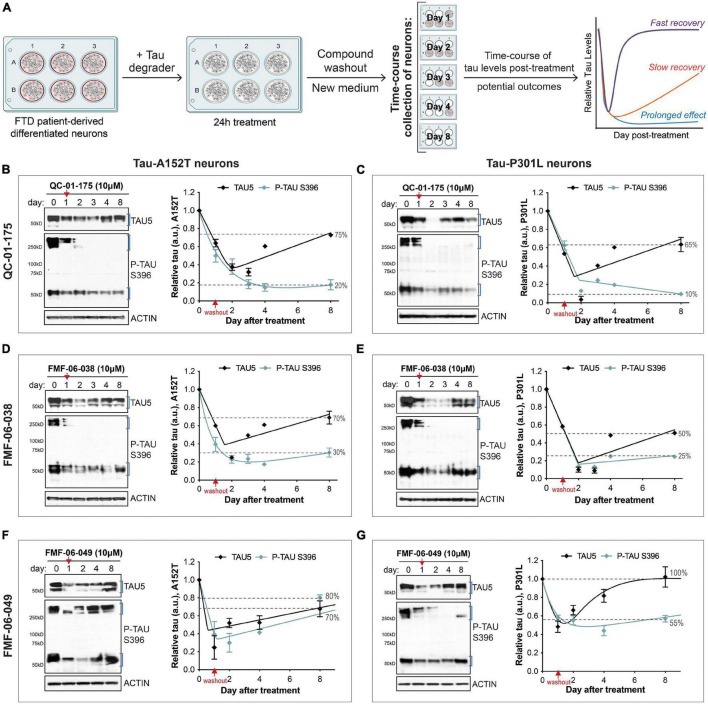
Single 24 h dose of each lead tau degrader has a prolonged effect on tau reduction. **(A)** Schematic of the assay implemented to measure effect of lead degraders (QC-01-175, FMF-06-038, FMF-06-049) on tau levels of FTD neurons (A152T or P301L) differentiated for 6 weeks. After 24 h incubation with each degrader and medium compound washout, neurons were analyzed over an 8-day time-course by western blot of tau and P-tau levels. Graph on the right illustrates the proposed possible outcomes for the trajectory of tau levels over time post-treatment. **(B–G)** FTD tau-A152T neurons **(B,D,F)** or tau-P301L **(C,E,G)** were treated with QC-01-175 **(B,C)**, FMF-06-038 **(D,E)** or FMF-06-049 **(F,G)** for 24 h, followed by compound washout (red arrows). Total tau (TAU5), P-tau S396 and actin were measured by western blot over a period of 8 days post-treatment. Representative blots are shown, and graph data points represent mean densitometry (bands within brackets) ± SD for *n* = 2 biological replicates. Dotted lines highlight the% of total tau and P-tau in neurons after 8 days, relative to day 0/vehicle samples.

NPCs were cultured in 6-well (Fisher Scientific Corning) or 96-well flat bottom (Fisher Scientific Corning) plates coated with poly-ornithine (20 μg/mL in water, Sigma) and laminin (5 μg/mL in PBS, Sigma), referred to as POL-coated plates. Culture medium was DMEM/F12-B27 [70% DMEM (Gibco), 30% Ham’s-F12 (Fisher Scientific Corning), 2% B27 (Gibco), 1% penicillin-streptomycin (Gibco)], supplemented with EGF (20 ng/mL, Sigma), FGF (20 ng/mL, Stemgent) and heparin (5 μg/mL, Sigma) for NPC proliferation. For NPC differentiation over the course of several weeks (6–8 weeks, experiment-dependent), cells were plated at an average density of 75,000 cells/cm^2^ and growth factors were omitted from the medium, which was exchanged twice per week (Silva et al., 2016). Compound treatment in 6-well plate format was performed in 2 mL medium volume by removing 1 mL of conditioned medium from the culture and adding 1 mL of new medium pre-mixed with the compound at the appropriate 2X concentration, followed by incubation at 37°C for the designated time. Compound treatment in 96-well plates was performed in 100 μL medium by adding compound directly to each well, followed by incubation at 37°C. As a control, neurons were also treated with vehicle alone, i.e., DMSO at 0.1% v/v, and this corresponds to 0 μM degrader treatment in Figures 1**–**5. Compounds were kept as 10 mM stock concentrations and serial dilutions were prepared before each experiment in 10-fold dilutions so that the same volume (and % DMSO) was added per well of cells, independent of degrader final concentration.

### Neuronal Viability Assay

NPCs were plated at an average density of 75,000 cells/cm^2^ in black 96-well plates with a clear flat bottom (Fisher Scientific Corning), differentiated for 6 weeks, and treated with compound for 24 h. Viability was measured with the alamarBlue HS cell viability reagent (Thermo Fisher Scientific) at 1:10 dilution in 100 μL cell medium, after 4 h incubation at 37°C, according to manufacturer’s instructions. Absorbance at 570 nm was measured in a spectrophotometric microplate reader, Spectramax Plus 384 (Molecular Devices), calculations were performed in Microsoft Excel (v.16.52) and graphs were plotted in GraphPad Prism 9 (v.9.2.0).

### Cell Lysis and Western Blot Analysis

NPCs were differentiated in 6-well plates at an average density of 75,000 cells/cm^2^. Neurons were washed in DPBS (Corning), lifted into suspension by scrapping, transferred to eppendorf tubes, and spun down at 3,000 × *g* for 5 min. Cell pellets were lysed in RIPA buffer (Boston Bio-Products) supplemented with 2% SDS (Sigma), 1% Halt Protease/Phosphatase inhibitors (Thermo Fisher Scientific), 1:5,000 Benzonase (Sigma), and 10 mM DTT (New England BioLabs), for 15 min at room temperature. Lysates were centrifugated at 20,000 × *g* for 20 min and the supernatants were transferred to new eppendorf tubes for analysis. Protein concentration quantification was performed using the Pierce BCA Protein Assay kit (Thermo Fisher Scientific). For western blot, 10 μg of total protein per well and in SDS blue loading buffer (New England Biolabs), were analyzed by SDS-PAGE. Alternatively, cell lysis was performed directly in 8X the cell pellet volume with SDS sample loading buffer (New England Biolabs) and boiled for 15 min prior to SDS-PAGE. Electrophoresis was performed with the Novex NuPAGE SDS-PAGE Gel System (Invitrogen). Proteins were transferred from the gel onto PVDF membranes (EMD Millipore) using standard procedures. Membranes were blocked in 5% BSA (Sigma) in Tris-buffered saline with Tween-20 (TBST, Boston Bio-Products), incubated overnight with primary antibody in 5% BSA-TBST at 4°C, followed by incubation with the corresponding HRP-linked secondary antibody at 1:4,000 dilution (Cell Signaling Technology). Blots were developed with SuperSignal West Pico Chemiluminescent Substrate (Thermo Fisher Scientific) according to manufacturer’s instructions, exposed to autoradiographic films (LabScientific by Thermo Fisher Scientific), and scanned on an Epson Perfection V800 Photo Scanner. Protein bands’ densitometry (pixel mean intensity in arbitrary units, a.u.) was measured with Adobe Photoshop 2021 (v.22.4.3) Histogram function and normalized to the respective internal control (β-ACTIN) band. Calculations were performed in Microsoft Excel (v.16.52), and graphs were plotted in GraphPad Prism 9 (v.9.2.0). The antibodies used were as follows: total tau TAU5 (Invitrogen AHB0042), P-tau Ser396 (Invitrogen 44752G), P-tau Ser202/Thr205 or AT8 (Thermo Fisher Scientific MN1020), CRBN (ProteinTech 11435-1-AP), VHL (Santa Cruz sc-135657), CHIP (Cell Signaling Technology 2080), GAPDH (Abcam ab8245), and β-ACTIN (Sigma A1978).

### Tau ELISA

NPCs were differentiated in 96-well plates at an average density of 100,000 cells/cm^2^ for 6 weeks, at which time neurons were lysed in an ELISA-compatible buffer (Invitrogen FNN0011) supplemented with 1 mM PMSF, protease inhibitors cocktail (Roche) and phosphatase inhibitors (Sigma) and incubated for 30 min on ice. Lysates were centrifugated at 18,000 × *g* at 4°C for 10 min and cleared lysates’ total protein concentration was determined with the Pierce BCA Protein Assay kit (Thermo Fisher Scientific). Tau ELISA assays were performed with the Human Total Tau ELISA Kit (Invitrogen) and the P-Tau[pS396] Human ELISA Kit (Invitrogen), according to manufacturer instructions. Spectrophotometric measures were done in a Spectramax Plus 384 (Molecular Devices) microplate reader. Microsoft Excel (v.16.52) was used for all calculations, and graphs and statistical analysis were done in GraphPad Prism 9 (v.9.2.0).

### Protein Solubility Analysis

For the solubility assays, cell lysis and protein fractionation based on detergent solubility were performed as previously described (Figure 2A; Guo and Lee, 2011; Kfoury et al., 2012; Silva et al., 2016). Briefly, higher solubility proteins (S fractions) were purified in 1% Triton buffer [1% Triton X-100 (Thermo Fisher Scientific), 1% Halt Protease/Phosphatase inhibitors (Thermo Fisher Scientific), 1:5,000 Benzonase (Sigma) and 10 mM DTT (New England BioLabs) in DPBS], whereas lower solubility pelleted proteins (P fractions) were resuspended in 5% SDS buffer [5% SDS (Sigma), 1% Halt Protease/Phosphatase inhibitors (Thermo Fisher Scientific), 1:5,000 Benzonase (Sigma) and 10 mM DTT (New England BioLabs) in RIPA buffer]. SDS-PAGE western blot was performed by loading 20 μg of each S-fraction and equal volume of the P-fraction onto pre-cast Tris-Acetate SDS-PAGE (Novex, Invitrogen). Western blot was performed as before. Densitometry values (pixel mean intensity in arbitrary units, a.u.) were measured with the Histogram function of Adobe Photoshop 2021 (v.22.4.3) and normalized to the respective GAPDH intensity in the S-fraction. Calculations were done in Microsoft Excel (v.16.52) and graphs were plotted in GraphPad Prism 9 (v.9.2.0).

### Time-Course Analysis of Tau Levels

Neurons were differentiated for 6 weeks (Figure 3A) and on day 0, vehicle alone (DMSO) or tau degrader compound were added to the neuronal cultures and incubated for 24 h at 37°C. On day 1, neuronal medium was replaced with new medium without compound, which is referred to as “compound washout.” At each time point after the 24 h treatment, one well of cells was sacrificed for lysis and protein analysis of tau by western blot as described above.

### Statistical Information

Graphed data represent mean values ± SD (standard deviation) or ± SEM (standard error of the mean), calculated using Microsoft Excel and GraphPad Prism. *P*-value < 0.05 was considered the threshold for statistical significance. *P*-value significance intervals (*) are provided within each figure legend, together with the statistical test performed for each experiment. *N*-values are indicated within figure legends and refer to biological replicates (NPC differentiation cultures independent setup and analysis, at different times), whereas technical replicates refer to the repeated analysis of the same samples. Derived statistics correspond to analysis of averaged values across biological replicates.

## Results

### Cereblon-Recruiting Molecular Series Dose-Effect on Tau of Frontotemporal Dementia Neuronal Models

We designed degrader compounds derived from the 5*H*-pyrido [4,3-*b*]indole scaffold of the tau PET tracer T807 (AV-1451), coupled to the CRBN ligand pomalidomide, and varying linker lengths and composition (Figures 1B–E). As the binding mode of T807 to misfolded tau in *ex vivo* human neurons was uncharacterized, we surveyed a broad range of linker lengths (Figure 1E).

To assess the cellular permeability of the synthesized molecules, we performed a cellular CRBN target engagement assay (Supplementary Figure 1A) that evaluates the molecules’ ability to compete with dBET6 for CRBN occupancy, as described in Donovan et al. (2020). In this *in vitro* assay, we observed low permeability for all analogs with a PEG linker, and modest permeability for QC-01-178, the only molecule with an alkyl linker (Figure 1E and Supplementary Figures 1B–J). Despite this result, since targeted protein degraders can affect degradation of their substrates at sub-stoichiometric cellular concentrations, we proceeded with testing the compound series in FTD patient iPSC-derived neuronal models to determine ability to change tau levels in *ex vivo* neurons.

We treated neurons derived from patients harboring the disease risk variant tau-A152T (Coppola et al., 2012; Lee et al., 2013; Decker et al., 2016; Maeda et al., 2016; Pir et al., 2016; Silva et al., 2016) or the pathogenic mutation tau-P301L (Hutton et al., 1998; Spillantini et al., 1998; Mirra et al., 1999; Rizzu et al., 1999; Silva et al., 2020) for 24 h with tau degrader at concentrations between 10 nM and 10 μM. We measured neuronal viability and observed that all compounds were non-toxic under the experimental conditions (Figure 1F). We observed potent degradation of P-tau S396, particularly of high MW oligomeric tau (Figure 1G and Supplementary Figure 2A, brackets), in both neuronal lines, for compounds with PEG_2_ (QC-01-175) and PEG_3_ (QC-01-208) linker lengths and at concentrations as low as 100 nM (Figures 1I,K and Supplementary Figure 2C). In parallel, there was also a dose-dependent reduction in total tau (TAU5 antibody). Significant loss of activity in the A152T neurons was observed for compounds with shortest linker lengths (QC-01-179, QC-01-178—Figures 1H,O), and complete loss of activity was observed for longer linker lengths (QC-01-176, QC-02-001, QC-02-004—Figures 1L–N). These trends in potency were mirrored in P301L neurons (Supplementary Figures 2B,F–I). As previously described (Silva et al., 2019), we also generated a negative control compound, QC-03-075, by replacing the glutarimide in pomalidomide with a δ-lactam ring that abrogates CRBN binding capacity, and as expected this compound showed no activity in either neuronal model (Figure 1J and Supplementary Figure 2D). Altogether, QC-01-175 concentration-dependent effect on tau, validation in two distinct neuronal models, and its superior potency and low MW relative to other active analogs, led to QC-01-175 selection as the lead compound of this series for further study (Silva et al., 2019).

### Optimization of Linker Attachment Chemistry and Improvement of Tau Degraders Activity

To further investigate the SAR for CRBN-recruiting degraders, we synthesized degraders with the ligand thalidomide (Figures 2A–C) *via* an aryl ether attachment chemistry that has been reported to reduce IMiD off-target degradation and improve cell permeability (Figure 2D; Brand et al., 2019). We incorporated the linkers which were found to enable tau degradation in at least one neuronal tauopathy model in the first QC-series and excluded the longer linker lengths of the inactive analogs. Cellular CRBN target engagement assays revealed that the new series of degraders (FMF-06-X) also displayed poor cell permeability in the *in vitro* cellular assay (Supplementary Figures 3A–F). Nonetheless, in tau-A152T neurons (Figures 2E–J) and in tau-P301L neurons (Supplementary Figures 3H–M) treated for 24 h with doses between 1 nM and 10 μM, we observed comparable potency for the anilino analogs FMF-06-038 (QC-01-175 analog) and FMF-06-050 (QC-01-208 analog), without loss of cellular viability (Supplementary Figure 3G). Moreover, up to 10-fold improvement in degradation potency of P-tau S396 and total tau was observed with the most active analog FMF-06-049, which harbors a six-carbon linker, with ∼60% total reduction in protein in A152T neurons (Figure 2J). When A152T neurons were treated for just 4 h, similar trends were observed (Figures 2K–P), with degradation of total tau and P-tau S396 up to 60–80% by FMF-06-038 (Figure 2L) and FMF-06-049 (Figure 2P). In fact, at 4 h FMF-06-049 reduced tau by almost 50% at the 10 nM dose. On a technical note, western blots in Figures 2E,K reveal intermediate oligomeric P-tau species between 60 and 150 kDa that were not detected in Figure 1G. This has to do with technical artifacts of western blot reagents and detection sensitivity, as well as possible variation across temporal replication of neuronal cultures. Most importantly, the species being compared across experiments, i.e., monomeric tau of ∼50 kDa and high MW oligomeric tau of ≥ 250 kDa, are consistently detected at similar intensity across experiments. In P301L neurons, FMF-06-049 was also the most potent analog, however, lower activity was observed in these neurons, with 100 nM compound required to effect significant tau degradation at 24 h (Supplementary Figure 3M). Robust degradation was also observed at 1 μM with FMF-06-038 in P301L neurons (Supplementary Figure 3I).

For the new CRBN-recruiting degrader series, the two molecules FMF-06-038 and FMF-06-049 showed significant and improved impact on tau clearance at the 10–100 nM dose range and across two tauopathy neuronal models, including with a shorter treatment of 4 h.

### Von Hippel-Lindau-Recruiting Tau Degraders Show Inferior Efficacy in Human Neurons

The choice of the recruited E3 ligase can impact the potency and specificity of degrader molecules and the extent of degradation of the POI. As a continued effort to identify optimal protein degraders for tau in human neurons, we investigated the potential of VHL-recruiting tau degraders (Figures 3A–E; Naro et al., 2020; Ishida and Ciulli, 2021; Wang et al., 2021). With evidence that the E3 ligase VHL, as for CRBN, is expressed in human iPSC-derived neurons (Supplementary Figures 4A–D), we synthesized molecules derived from the 5*H*-pyrido[4,3-*b*]indole scaffold of the tau ligand T807 (Figure 3B), coupled to a VHL ligand (Figure 3C; Raina et al., 2016) and a range of linker lengths (Figures 3D,E).

A VHL cellular target engagement assay (Supplementary Figures 4E–L) revealed that these analogs also had poor cellular permeability, with detectable activity only for the alkyl-chain linker analog FMF-06-066-1 (Supplementary Figure 4L). To examine the effects of varying the E3 ligase on tau degradation, these molecules were tested by both western blot and ELISA in A152T and P301L neurons (24 h), to determine changes on tau protein levels. For its increased sensitivity and quantitative power, ELISA results are shown here (Figures 3F–L and Supplementary Figures 5A–G). Some molecules showed activity, promoting up to 50% reduction in P-tau and/or total tau levels at specific concentrations (e.g., 100 nM FMF-05-129-2 and 10 nM FMF-06-066; Figures 3G,L), particularly in the P301L line (e.g., 10 nM FMF-05-129-5, Supplementary Figure 5F). However, all analogs failed to show an evident concentration-dependent effect and exhibited lower degradation efficiency than the CRBN-recruiting series. Nonetheless, treatment with the FMF-06-066-1 diastereomer FMF-06-064-1 (Figure 3H and Supplementary Figure 5C), which can no longer engage VHL, rescued this activity, confirming the proposed mode of action (Figure 3A).

The results with VHL-recruiting degraders suggest that in human neurons, specifically in FTD patient iPSC-derived *ex vivo* neurons, CRBN has higher activity toward tau ubiquitination for proteasome degradation (Silva et al., 2019) and might be a better therapeutic target than VHL. This may have to do with the VHL E3 ligase system being potentially “affected” or disrupted in disease. As we show in Supplementary Figures 4A–D, for two WT and two mutant tau lines, there is a significant increase in VHL levels in patient mutant tau neurons relative to control tau WT neurons (Supplementary Figure 4C) and coincidental with tau accumulation (Supplementary Figure 4D). In contrast, for CRBN, the protein steady-state levels are constant across genotypes and independent of tau levels (Supplementary Figure 4B).

### Degraders Preferentially Target Insoluble Tau

Our CRBN-recruiting bifunctional degraders were designed based on the PET imaging probe T807, which binds hyperphosphorylated tau *in vivo* in a conformation dependent manner, with highest affinity for AD-like paired helical filaments (PHF) (Jones et al., 2018) and higher uptake in brain regions with significant tau burden. In this context, and to gain some insight into the tau species targeted for degradation in patient-derived *ex vivo* neurons (Figure 1A), we evaluated the effect of degraders on tau species of different solubility. For this study we focused on the first-generation molecule QC-01-175 and the two improved derivative molecules FMF-06-038 and FMF-06-049. In addition, we focused on the tau-P301L neuronal model as a representative of tauopathy pathology associated with accumulation of insoluble species in the brain (oligomers, aggregates, and higher order fibrils and tangles) (Nacharaju et al., 1999; Barghorn et al., 2000; Ramsden et al., 2005; Kimura et al., 2010).

We treated neurons for 24 h at the concentration leading to maximum tau reduction (10 μM) and at the half maximal effective concentration (EC_50_, 100 nM), and proceeded with cellular lysis and protein fractionation based on differential detergent solubility (Figure 4A; Guo and Lee, 2011; Kfoury et al., 2012). Western blot analysis of the soluble (S) and insoluble-pellet (P) protein fractions upon treatment, and relative to vehicle controls, allowed us to assess the effect of each degrader on soluble and insoluble forms of total tau (TAU5), P-tau S396 and P-tau S202/T205 (AT8), the later one also observed in patient brain pathology (Figures 4B,D; Braak et al., 1994, 2011; Goedert et al., 1995). Notably, we observed accentuated and preferential degradation of insoluble tau (P) fractions by all the degraders (Figures 4C,E,F), with a reduction in protein by 80–100% at the highest concentration, including the high MW oligomeric P-tau. Whereas QC-01-175 and FMF-06-049 also led to a 40–50% decrease in soluble (S) tau and P-tau (Figures 4C,F), FMF-06-038 showed specificity only for insoluble tau (Figure 4E).

These results reveal that the second-generation molecule FMF-06-049 has the highest activity for tau degradation in human *ex vivo* neurons, with 100% clearance of insoluble tau (Figure 4F). Importantly, the observed degrader higher specificity toward insoluble tau reinforces the strategy employed in the chemistry design and demonstrates that the T807 scaffold ligand conformation-dependent affinity is likely conserved in the degrader function in *ex vivo* human neurons (Figure 1A).

### Kinetics of Tau Degradation in Frontotemporal Dementia Neurons Reveal a Prolonged Effect

In addition to 24 h-treatments followed by immediate measure of tau levels, we were interested in evaluating the longer-term effect of degrader treatment in FTD neurons (Figure 5A). We considered three potential scenarios for changes in tau levels beyond the 24 h: (*i)* tau quickly returns to basal levels (“fast recovery”), (*ii)* tau levels continue to decrease followed by a slow recovery to basal levels (“slow recovery”), or (*iii)* tau levels not only decrease further but are maintained at low levels for a period of time as a consequence of the degrader molecule catalytic effect (“prolonged effect”) (Sun et al., 2019; Gao et al., 2020; Mares et al., 2020). We investigated these scenarios in both A152T and P301L neuronal models, treated with QC-01-175, FMF-06-038 or FMF-06-049 for 24 h at 10 μM (maximum tau reduction, Figure 1I and Supplementary Figures 2C,F,J, 3I,M). After incubation with the degrader, we performed compound washout, i.e., we washed the cells and added new culture medium without compound (Figure 5A). Neurons were collected over a time-course between 1 and 8 days, and tau and P-tau levels were measured by western blot (Figures 3B–G). Day zero corresponds to vehicle-treated samples. After 24 h treatment (day 1), tau was reduced by 40–60% with the expected variability between degraders and neuronal lines (Figures 5B–G graphs’ red arrow). Notably, after QC-01-175 and FMF-06-038 media washout (Figures 5B–E), the levels of total tau and P-tau S396 continued to decrease for three more days, bringing tau levels further down to only 10–40% of vehicle-treated samples (dashed lines, Figures 5B–E). While we observed more rapid recovery of monomeric tau, there was a remarkable prolonged loss of P-tau S396 to 10–25% of vehicle control, with no detection of high MW oligomeric P-tau up to day 8. Conversely, FMF-06-049 showed the lowest levels of tau at 24 h-post treatment and a quicker recovery to basal levels in the 8-day time-course (Figures 5F,G).

Altogether, these data suggest that the second-generation degraders FMF-06-038 and FMF-06-049 show improved activity for tau clearance with high specificity for insoluble protein, and promote prolonged reduction in tau levels. Differences in the ranking between these two molecules are assay-dependent (compare Figures 4, 5) and suggest that, although improved, the molecules have different levels of specificity/affinity for insoluble tau as well as distinct catalytic activity properties.

## Discussion

Cellular models of misfolded tau are challenging to generate due to complex protein post-translational modifications, multiple isoforms expression, structural features induced by disease-associated tau variants and mutations, and possible contributions from the genetic background and physiological context. In this study, we harnessed FTD patient iPSC-derived neurons expressing the variant A152T or the mutation P301L (Silva et al., 2016, 2020), to ensure presence of misfolded conformers that would be recognized by the tau ligand in the degrader (Figures 1A,B). Nonetheless, one of the characteristics of iPSC-derived neurons is lack of maturity and expression of all tau isoforms found in the adult human brain (Silva et al., 2016; Sato et al., 2018), which may cause some differences in tau conformers found in the adult, aging brain and in the cellular system. Even so, patient-specific iPSC-derived neurons reveal tau phenotypes that are genotype dependent and disease relevant, including with 4R specific mutations like P301L (Silva et al., 2016, 2020; Seo et al., 2017). Increasing evidence show that early alterations in brain development and homeostasis contribute to later manifestation of neurodegenerative diseases, encouraging the use of iPSC-derived systems to study disease (Faravelli et al., 2020; Silva and Haggarty, 2020; de Rus Jacquet et al., 2021). Therefore, identification of early molecular changes and early tau toxic conformers that precede late-stage pathology and cell death, represent therapeutic targets most relevant when intervention will likely have most impact.

The principle aim of this study was to determine the SAR for different series of tau degraders, culminating in the discovery of the previously disclosed QC-01-175 and here enhanced analogs (Silva et al., 2020). We observed potent degradation with our optimized analogs, despite minimal E3-ligase occupancy measured by *in vitro* cellular target engagement assays, indicating low cell permeability of our compounds. Compound potency also varied between the two different neuronal models in this study, i.e., the risk variant tau-A152T and the autosomal dominant pathogenic mutation tau-P301L, consistent with their distinct predicted pathological characteristics and aberrant tau species formed and, therefore, potentially different affinities for the tau ligand in the degrader. Here, we prioritized potent hits that showed robust tau degradation across both models. By utilizing models derived from different patients, we were able to gain further insight into how our compounds may perform in different disease contexts, which is relevant for tauopathies with high levels of heterogeneity across the patient population. Future studies will be dedicated to understanding the specificity of the degraders toward specific tau misfolded conformers, mutant protein, and post-translationally modified tau. It is unknown, for patient-specific neurons, and for most human pathological studies, what the ratio of mutant vs. WT tau protein that populate the aberrant tau species. Considering that these are heterozygous neurons, and that the degrader can reduce tau by more than 50% (e.g., Figure 1I), one can speculate that the aberrant species targeted for degradation, in these specific models, might include WT protein that has been recruited into a misfolded conformation.

Following degrader treatment and targeted tau degradation, new aberrant tau must be not only transcribed and translated, but also must re-populate the misfolded species observed in tauopathy patient-derived iPSC neurons. Therefore, we hypothesized that misfolded tau levels would display slow recovery for re-accumulation following degradation. Consistently, we observed slow recovery of total tau (TAU5) levels, and prolonged reduction of particularly P-tau high MW oligomers out to 8 days following compound washout. These results indicate that tau degraders have long-lived effects intracellularly that serve to reduce misfolded tau and delay further accumulation, possibly even well after the compound is cleared from the cell. This highlights a tremendous benefit of a targeted tau degradation approach as a therapeutic intervention. Importantly, these effects would likely be difficult to observe in cellular model systems where tau heterologous overexpression and aggregation deviate from physiological conditions and highlight the advantages of patient-derived models for informing on small molecule specificity and duration of action.

## Data Availability Statement

The original contributions presented in the study are included in the article/Supplementary Material, further inquiries can be directed to the corresponding author/s.

## Author Contributions

MS, GN, KD, QC, BB, RN, and FF performed the experiments and analyzed the data. EF, NG, and SH supervised the project and analyzed the data. FF and MS wrote the manuscript with edits from all authors. All authors read and approved the manuscript.

## Conflict of Interest

MS, FF, QC, NG, and SH are co-inventors on a patent covering the molecules disclosed in this publication (WO/2019/014429). MS is a consultant to Casma Therapeutics. KD is a consultant to Kronos Bio. EF is a founder, SAB member, and equity holder in Civetta Therapeutics, Jengu Therapeutics (board member), Neomorph Inc., and Proximity Therapeutics; an equity holder of C4 Therapeutics; and a consultant to Novartis, AbbVie, Sanofi, Deerfield, Avilar Pfizer, and EcoR1. The Fischer lab receives or has received research funding from Novartis, Astellas, Ajax, and Deerfield. NG is a founder, science advisory board member (SAB), and equity holder at Gatekeeper, Syros, Petra, C4, B2S, Voronoi, EoCys, Larkspur (board member), and Soltego (board member). The Gray lab receives or has received research funding from Novartis, Takeda, Astellas, Taiho, Jansen, Kinogen, Her2llc, Arbella, Deerfield, and Sanofi. FF is a founder and equity holder in Proximity Therapeutics, an SAB member and equity holder in Triana Therapeutics, and a consultant to RA Capital. SH is a member of the SAB and equity holder of Psy Therapeutics, Frequency Therapeutics, Sensorium Therapeutics, 4M Therapeutics, and Proximity Therapeutics and has received speaking fees from AstraZeneca, Amgen, Merck, and Syros. The Haggarty lab has received funding from the Tau Pipeline Enabling (T-PEP) Program (Alzheimer’s Association/Rainwater Foundation), Tau Consortium, F-Prime Biomedical Research Initiative, AstraZeneca, JW Pharmaceuticals, Lexicon Pharmaceuticals, Stealth Biotherapeutics, atai Life Sciences, and Compass Pathways. The remaining authors declare that the research was conducted in the absence of any commercial or financial relationships that could be construed as a potential conflict of interest.

## Correction note

A correction has been made to this article. Details can be found at: 10.3389/fncel.2026.1830139.

## Publisher’s Note

All claims expressed in this article are solely those of the authors and do not necessarily represent those of their affiliated organizations, or those of the publisher, the editors and the reviewers. Any product that may be evaluated in this article, or claim that may be made by its manufacturer, is not guaranteed or endorsed by the publisher.
