# Correction: Discovery and optimization of tau targeted protein degraders enabled by patient induced pluripotent stem cells-derived neuronal models of tauopathy

**DOI:** 10.3389/fncel.2026.1830139

**Published:** 2026-04-07

**Authors:** M. Catarina Silva, Ghata Nandi, Katherine A. Donovan, Quan Cai, Bethany C. Berry, Radoslaw P. Nowak, Eric S. Fischer, Nathanael S. Gray, Fleur M. Ferguson, Stephen J. Haggarty

**Affiliations:** 1 Chemical Neurobiology Laboratory, Department of Neurology and Psychiatry, Center for Genomic Medicine, Massachusetts General Hospital, Boston, MA, United States; 2 Department of Neurology, Harvard Medical School, Boston, MA, United States; 3 Department of Cancer Biology, Dana-Farber Cancer Institute, Boston, MA, United States; 4 Department of Biological Chemistry and Molecular Pharmacology, Harvard Medical School, Boston, MA, United States

**Keywords:** tau, structure-activity relationships, targeted protein degradation, PROTAC, human stem cells, human neuronal models, frontotemporal dementia

There was a mistake in Figure 1G as published.

**Figure 1:**
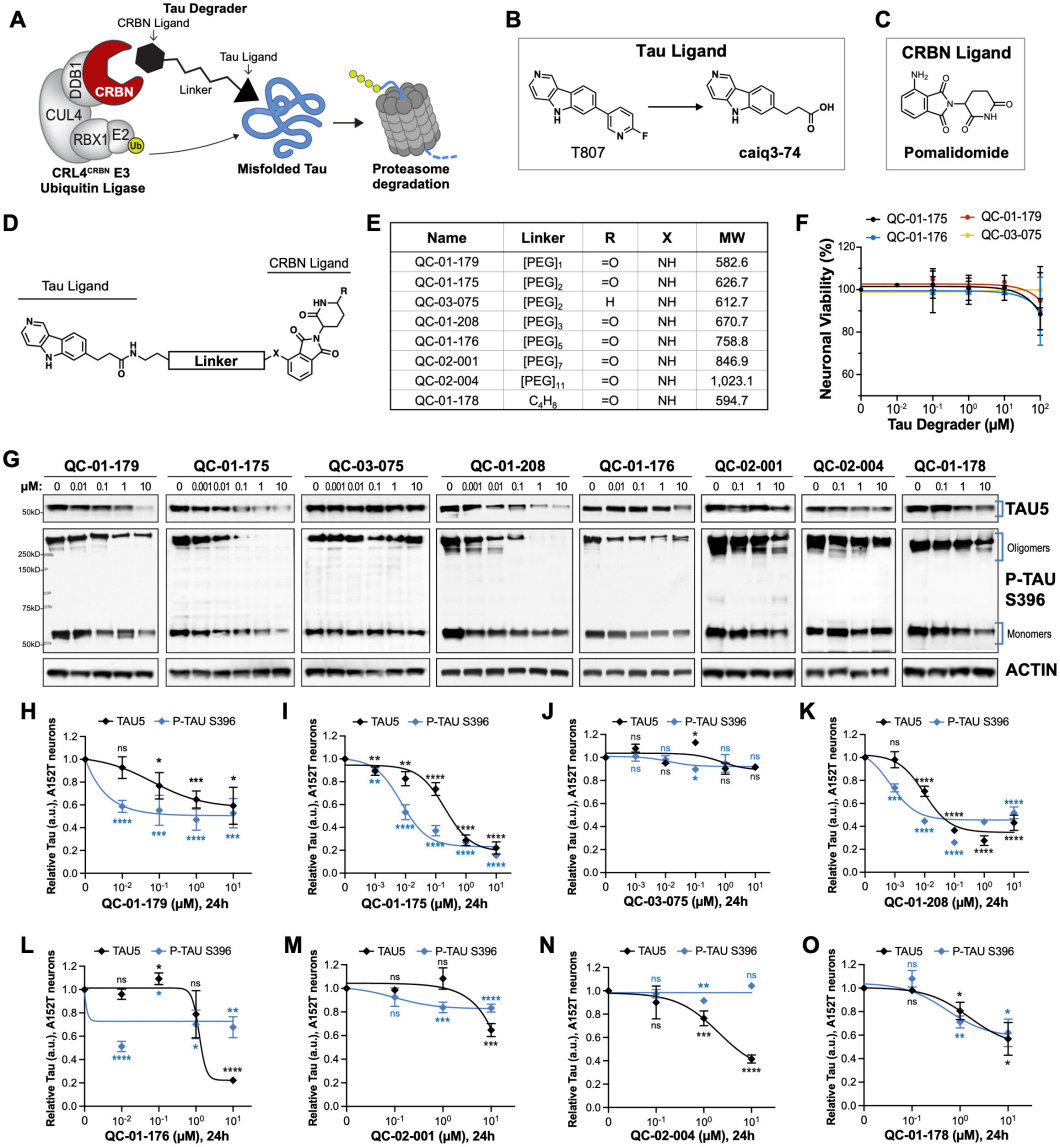
Design strategy for CRBN-recruiting degraders and concentration effect on tau and P-tau of human FTD neurons. **(A)** Working model for hetero-bifunctional tau degraders designed to preferentially recognize pathological forms of tau (misfolded protein) and simultaneously engage with CRBN (CRL4^*CRBN*^ E3 ubiquitin ligase complex). Formation of the ternary complex is expected to enhance tau ubiquitination and degradation by the proteasome. **(B–D)** Degraders were synthesized based on the T807 core scaffold for tau recognition **(B)**, a thalidomide analog as an E3 ligand (pomalidomide, **C**) for CRBN engagement, and a variable linker length and composition to maximize target clearance efficiency **(D)**. **(E)** Summary of the chemical properties of CRBN-recruiting degrader molecules of the QC-Series. **(F)** Neuronal viability of tau-A152T neurons at 6 weeks of differentiation treated with representative degraders of the series for 24 h. Viability is shown relative to vehicle-treated neurons (100%) and each data point represents the mean% viability ± SD, *n* = 3. **(G–O)** Concentration effect of degraders on tau protein of A152T neurons (6-week differentiated) by analysis of total tau (TAU5) and P-tau S396 levels upon treatment for 24 h. Representative western blots are shown **(G)** and brackets indicate bands quantified for TAU5 and P-tau S396 densitometry **(H–O)**. Data points represent mean densitometry normalized to actin and relative to vehicle ± SEM (*n* ≥ 3). Data points for QC-01-175 **(I)** and QC-03-075 **(J)** include values from new biological replicates averaged with analysis previously published (**Silva et al., 2019**). The 0 μM degrader data points correspond to vehicle alone (DMSO) control treatment. Statistics: 2-tailed unpaired Student's *t*-test for each concentration relative to vehicle; ^*ns*^*P* > 0.05, ^*^P < 0.05, ^**^P < 0.01, ^***^P < 0.001, ^****^P < 0.0001.

In the originally published figure, the actin loading-control lanes corresponding to QC-01-179 and QC-01-178 were displayed at reduced height and resolution, which compressed the bands and made adjacent lanes appear unusually similar. This presentation artifact created the appearance of potential lane duplication. The figure has now been regenerated from the original high-resolution TIFF files with confirmed correct lane selection and increased display height to improve visibility and distinguishability of the actin bands. This issue did not affect the data quantification or the resulting dose-response curves or text, as all analyses were performed using the original, unedited image files.

The corrected Figure 1G (within corrected Figure 1) appears below.

There was a mistake in Figure 2E as published.

**Figure 2:**
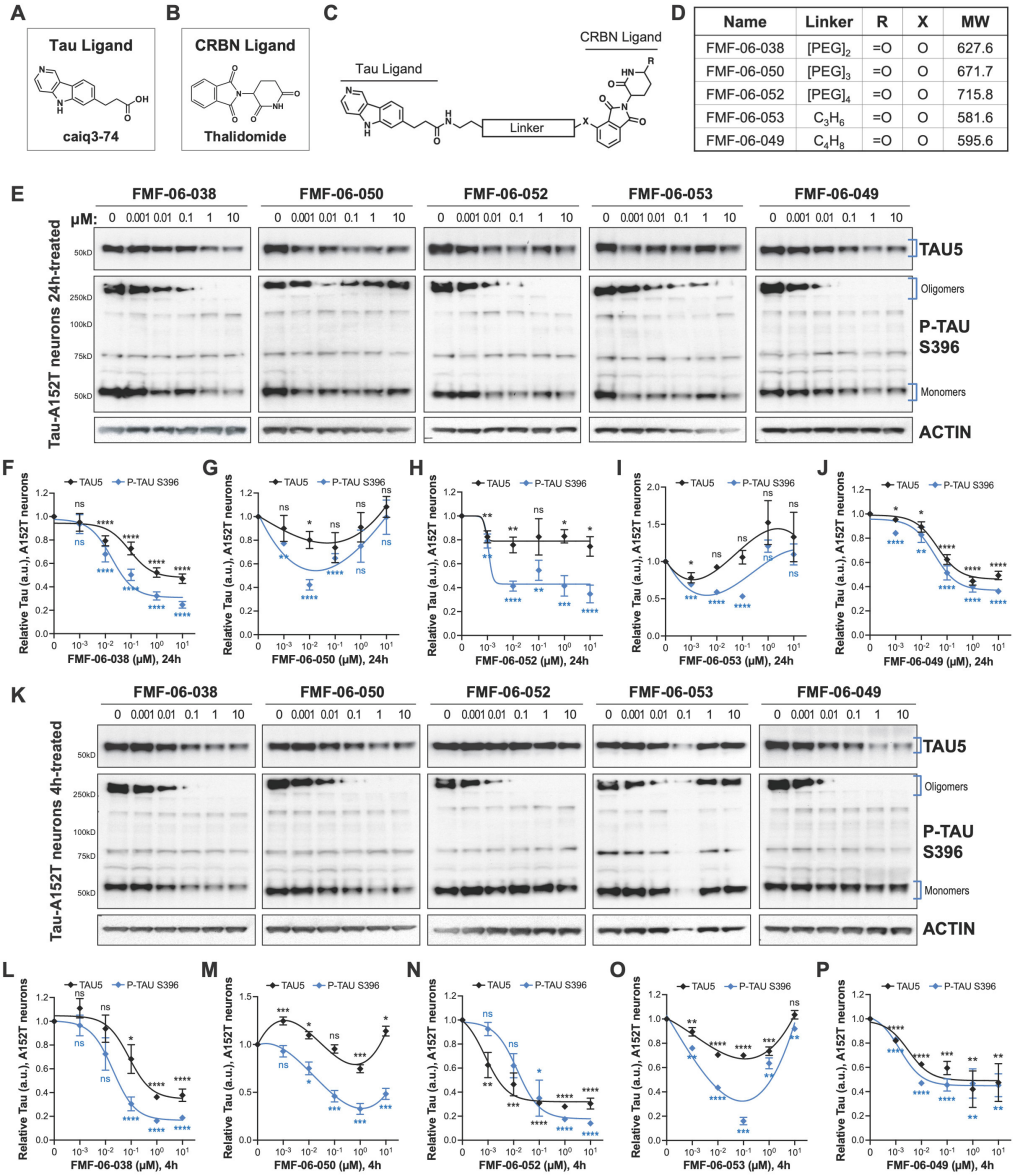
Characterization of second-generation CRBN-recruiting degraders (FMF-06-series) focusing on optimization of linker attachment chemistry. **(A–C)** The FMF-06-series was synthesized based on the T807 core scaffold for tau recognition **(A)**, thalidomide as the E3 ligand for CRBN engagement **(B)**, and a variable linker length and attachment composition **(C)**. **(D)** Summary of chemical properties of the FMF-06-Series. **(E–J)** Degraders' concentration effect on tau protein of A152T neurons (6-week differentiated) with analysis of total tau (TAU5) and P-tau S396 levels upon treatment for 24 h. Representative western blots are shown **(E)** and brackets indicate bands quantified for TAU5 and P-tau S396 densitometry **(F–J)**. Data points represent mean densitometry normalized to actin and relative to vehicle ± SEM (*n* = 3). **(K–P)** Degraders' concentration effect on tau protein levels of A152T neurons (6-week differentiated) after a 4 h treatment. Representative western blots are shown **(K)** for immunoprobing with TAU5, P-tau S396 and actin. Data points **(L–P)** represent mean densitometry normalized to actin and relative to vehicle ± SEM (*n* = 3). The 0 μM degrader data points correspond to vehicle alone (DMSO) control treatment. Statistics: 2-tailed unpaired Student's *t*-test for each concentration relative to vehicle; ^*ns*^*P* > 0.05, ^*^P < 0.05, ^**^P < 0.01, ^***^P < 0.001, ^****^P < 0.0001.

In the originally published Figure 2E, the actin loading-control lane corresponding to FMF-06-038 was accidentally duplicated from the sample FMF-06-052 during figure assembly. This error originated from an intermediate file in which the samples were arranged in a different order, leading to an inadvertent copy–paste duplication of the actin

lane. The panel has now been reassembled using the original scanned blot images to ensure correct lane assignment. Importantly, all quantifications were performed using the original, unedited image files; therefore, the densitometry analyses, resulting dose-response curves and text were not affected by this mistake.

The corrected Figure 2E (within **corrected Figure 2**) appears below.

The original version of this article has been updated.

