# Supplementary material for: Discovery and Optimization of Tau Targeted Protein Degraders Enabled by Patient Induced Pluripotent Stem Cells-Derived Neuronal Models of Tauopathy
Source: Front Cell Neurosci. 2022 Mar 3;16:801179. doi: 10.3389/fncel.2022.801179 (PMC8934437; doi:10.3389/fncel.2022.801179)
Supplement: Supplementary file 1 [file Data_Sheet_1.pdf]

Supplementary Information

General methods

Unless noted, reagents and solvents were obtained from commercial sources and were used without further purification. 1H NMR spectra were recorded on 500 MHz Bruker Avance III spectrometer, and chemical shifts are reported in parts per million (ppm, δ) downfield from tetramethylsilane (TMS). Coupling constants (J) are reported in Hz. Spin multiplicities are described as s (singlet), br (broad singlet), d (doublet), t (triplet), q (quartet), and m (multiplet). Mass spectra were obtained on a Waters Acquity UPLC. Preparative HPLC was performed on a Waters Sunfire C18 column (19 mm × 50 mm, 5 μM) using a gradient of 15−95% methanol in water containing 0.05% trifluoroacetic acid (TFA) over 22 min (28 min run time) at a flow rate of 20 mL/min. Assayed compounds were isolated and tested as TFA salts. Purities of assayed compounds were in all cases greater than 95%, as determined by reverse-phase HPLC analysis.

Scheme 1

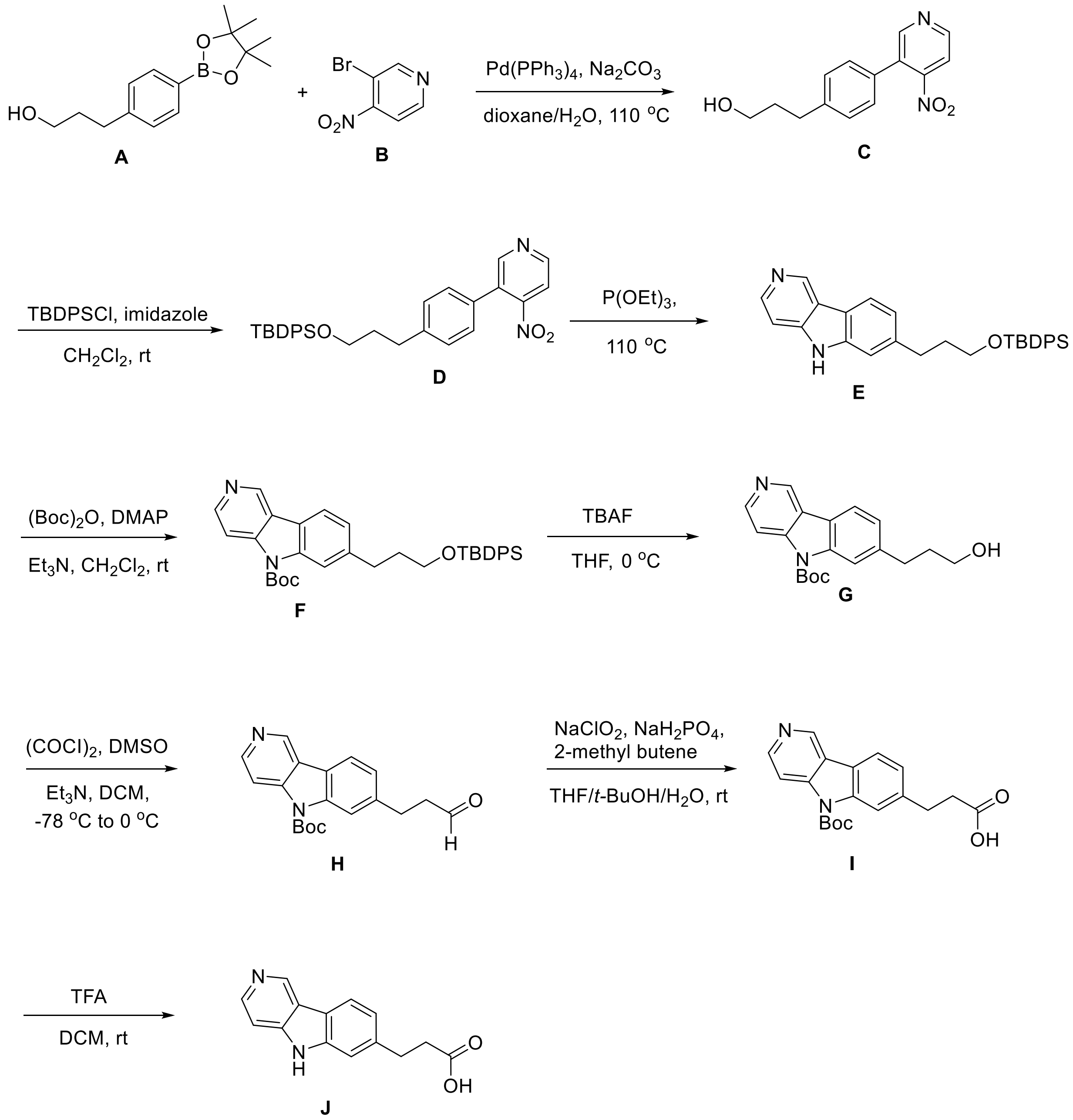

3-(4-(4-Nitropyridin-3-yl)phenyl)propan-1-ol (C): A solution of 3-(4-(4,4,5,5-tetramethyl-1,3,2-dioxaborolan-2-yl)phenyl)propan-1-ol (**A**) (1.65 g, 6.29 mmol), 3-bromo-4-nitropyridine (**B**) (1.16 g, 5.72 mmol), Na_2CO_3 (1.52 g, 14.3 mmol), and $Pd(PPh_3)_4$ (330 mg, 0.286 mmol) in 1,4-dioxane (40 mL) and H_2O (10 mL) was stirred at 110°C for 16 hrs before it was quenched with NH_4Cl (sat. aq., 100 mL). The resulting mixture was extracted with CH_2Cl_2 (3 × 80 mL), the combined organic phases were dried over anhydrous Na_2SO_4 and concentrated under reduced pressure. The residue was purified by flash column chromatography to give the title compound (950 mg, 3.68 mmol, 64% yield).

3-(4-(3-((*tert*-Butyldiphenylsilyl)oxy)propyl)phenyl)-4-nitropyridine (D): To a stirred solution of **C** (950 mg, 3.68 mmol) in CH_2Cl_2 (35 mL) at 25ºC was added imidazole (751 mg, 11.04 mmol) and TBDPSCl (2.01 g, 7.36 mmol). After stirring at this temperature for 3 hrs, the reaction was quenched with NH_4Cl (sat. aq., 100 mL). The resulting mixture was extracted with CH_2Cl_2 (2 × 50 mL), the combined organic phases were washed with brine (100 mL), dried over anhydrous Na_2SO_4 and concentrated under reduced pressure. The residue was purified by flash column chromatography to give the title compound (1.62 g, 3.26 mmol, 89% yield).

7-(3-((*tert*-Butyldiphenylsilyl)oxy)propyl)-5H-pyrido[4,3-*b*]indole (E): A solution of **D** (1.62 g, 3.26 mmol) in $P(OEt)_3$ (20 mL) was stirred at 110ºC for 3 hrs before it was concentrated under reduced pressure. The residue was purified by flash column chromatography to give the title compound (1.32 g, 2.84 mmol, 87% yield).

***tert*-Butyl 7-(3-((*tert*-butyldiphenylsilyl)oxy)propyl)-5H-pyrido[4,3-*b*]indole-5-carboxylate (F):** To a stirred solution of **E** (1.32 g, 2.84 mmol) and DMAP (213 mg, 1.75 mmol) in CH_2Cl_2 (30 mL) at 25ºC was added Et_3N (1.06 g, 10.47 mmol) and $(Boc)_2O$ (1.52 g, 6.98 mmol). After stirring at this temperature for 2 hrs, the reaction was quenched with NH_4Cl (sat. aq., 100 mL). The resulting mixture was extracted with CH_2Cl_2 (2 × 50 mL), the combined organic phases were washed with brine (100 mL), dried over anhydrous Na_2SO_4 and concentrated under reduced pressure. The residue was purified by flash column chromatography to give the title compound (1.42 g, 2.52 mmol, 89% yield).

***tert*-Butyl 7-(3-hydroxypropyl)-5H-pyrido[4,3-*b*]indole-5-carboxylate (G):** To a stirred solution of **F** (1.42 g, 2.52 mmol) in THF (25 mL) at 0ºC was added TBAF (1.0 M in THF, 3.8 mL, 3.8 mmol) dropwise. After stirring at this temperature for 2 hrs, the reaction was quenched with acetic acid (0.2 mL). The mixture was concentrated under reduced pressure. The residue was purified by flash column chromatography to give the title compound (670 mg, 2.05 mmol, 81% yield).

***tert*-b**utyl 7-(3-oxopropyl)-5H-pyrido[4,3-*b*]indole-5-carboxylate (H):** To a stirred solution of DMSO (1.30 g, 16.6 mmol) in CH_2Cl_2 (7 mL) at -78ºC was added oxalyl chloride (783 mg, 6.16 mmol) in CH_2Cl_2 (6 mL) dropwise. After stirring at this temperature for 0.5 hrs, a solution of **G** (670 mg, 2.05 mmol) in CH_2Cl_2 (6 mL) was added dropwise. The mixture was stirred at this temperature for 2 hrs followed by the addition of Et_3N (1.035 g, 10.25 mmol) dropwise. The reaction mixture was slowly warmed to 0ºC over 1 h and was quenched with NH_4Cl (sat. aq., 30 mL). The resulting mixture was extracted with CH_2Cl_2 (2 × 30 mL), the combined organic phases were washed with brine (100 mL), dried over anhydrous Na_2SO_4 and concentrated under reduced pressure to provide the title compound. The crude product of **H** was used in the next step without further purification.

3-(5-(*tert*-Butoxycarbonyl)-5H-pyrido[4,3-*b*]indol-7-yl)propanoic acid (**I**): To a stirred solution of the above residue of **H** in THF (10 mL), *t*-BuOH (5 mL) and H_2O (5 mL) at 25ºC was added NaH_2PO_4•H_2O

(2.55 g, 18.5 mmol), 2-methyl-butene (5 mL) and sodium chlorite (1.64 g, 18.5 mmol). After stirring at this temperature for 2 hrs, the reaction was diluted with H_2O (50 mL). The resulting mixture was extracted with CH_2Cl_2 (2 × 50 mL), the combined organic phases were dried over anhydrous Na_2SO_4 and concentrated under reduced pressure to provide the title compound. The crude product of **I** was used in the next step without further purification.

3-(5H-Pyrido[4,3-*b*]indol-7-yl)propanoic acid (**J**): A solution of the crude product of **I** in CH_2Cl_2 (12 mL) and TFA (6 mL) was stirred at 25ºC for 12 hrs before it was concentrated under reduced pressure. The residue was dissolved in NaOH (0.5 M, aq., 20 mL) and was extracted with CH_2Cl_2 (4 × 15 mL). The water phase was added HCl (aq., 1.0 M) dropwise to adjust the pH to 6-7. The resulting mixture was extracted with $CHCl_3$/*i*-PrOH (4/1, 3 × 30 mL), the combined organic phases were dried over anhydrous Na_2SO_4 and concentrated under reduced pressure to afford the title compound (357 mg, 1.48 mmol, 73% yield over 3 steps).

N-(6-((2-(2,6-Dioxopiperidin-3-yl)-1,3-dioxoisoindolin-4-yl)amino)hexyl)-3-(5H-pyrido[4,3-*b*]indol-7-yl)propanamide (1; QC-01-178))

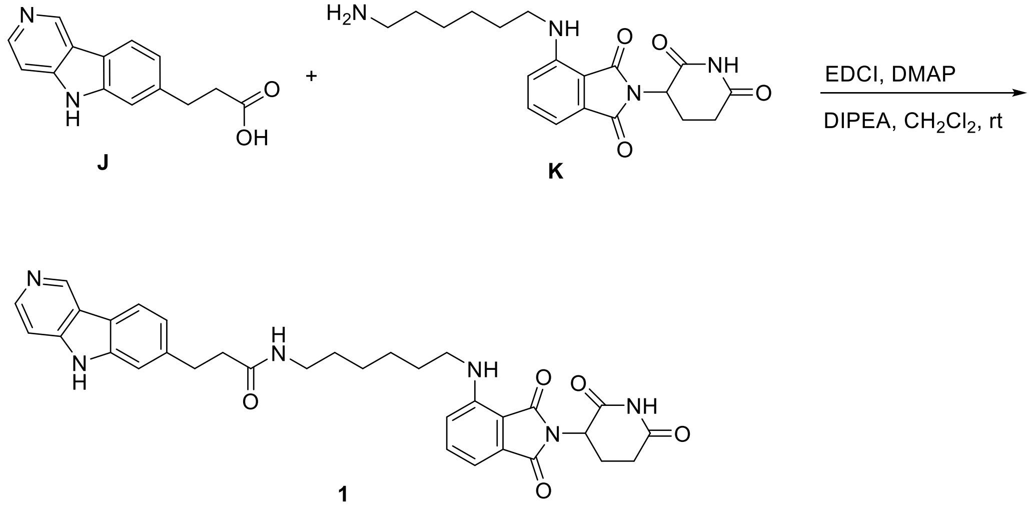

N-(6-((2-(2,6-Dioxopiperidin-3-yl)-1,3-dioxoisoindolin-4-yl)amino)hexyl)-3-(5H-pyrido[4,3-*b*]indol-7-yl)propanamide (1): To a stirred solution of carboxylic acid **J** (6.8 mg, 0.02 mmol), EDCI (11.5 mg, 0.06 mmol), DMAP (2.2 mg, 0.02 mmol) and DIPEA (14.3 mg, 0.12 mmol) in CH_2Cl_2 (0.6 mL) at 25ºC was added 4-((6-aminohexyl)amino)-2-(2,6-dioxopiperidin-3-yl)isoindoline-1,3-dione (**K**) (9.7 mg, 0.26 mmol). The resulting reaction mixture was stirred at this temperature for 4 hrs, and then concentrated under reduced pressure. The residue was purified by reverse-phase HPLC to give the title compound as the TFA salt (9.6 mg, 0.014 mmol, 70% yield). ^1H NMR (500 MHz, DMSO) δ 13.03 (s, 1H), 11.10 (s, 1H), 9.68 (s, 1H), 8.61 (d, *J* = 6.8 Hz, 1H), 8.33 (d, *J* = 8.1 Hz, 1H), 7.96 (d, *J* = 6.7 Hz, 1H), 7.80 (t, *J* = 5.6 Hz, 1H),

7.59 (s, 1H), 7.57 (dd, *J* = 8.5, 7.2 Hz, 1H), 7.36 (dd, *J* = 8.2, 1.0 Hz, 1H), 7.03 (d, *J* = 3.9 Hz, 1H), 7.02 (d, *J* = 2.3 Hz, 1H), 6.46 (t, *J* = 5.1 Hz, 1H), 5.05 (dd, *J* = 12.8, 5.4 Hz, 1H), 3.20 (dd, *J* = 12.4, 6.5 Hz, 2H), 3.06 – 3.00 (m, 4H), 2.89 (ddd, *J* = 17.0, 13.9, 5.4 Hz, 1H), 2.66 – 2.52 (m, 2H), 2.48 (t, *J* = 7.6 Hz, 2H), 2.06 – 2.01 (m, 1H), 1.49 – 1.43 (m, 2H), 1.37 – 1.31 (m, 2H), 1.29 – 1.23 (m, 2H), 1.22 – 1.16 (m, 2H).

Example compounds **2-7** were prepared in an analogous manner to compound **1**, employing the corresponding amine starting materials and carboxylic acid **J**.

	**Structure/Name**	**Characterization**
**2**	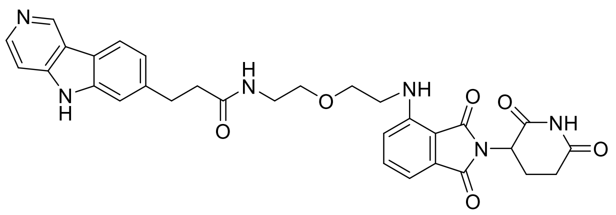<br>**QC-01-179**<br>N-(2-(2-((2-(2,6-dioxopiperidin-3-yl)-1,3-dioxoisoindolin-4-yl)amino)ethoxy)ethyl)-3-(5H-pyrido[4,3-b]indol-7-yl)propanamide	^{1}H NMR (500 MHz, DMSO) δ 13.00 (s, 1H), 11.08 (s, 1H), 9.65 (s, 1H), 8.61 (d, *J* = 6.8 Hz, 1H), 8.31 (d, *J* = 8.1 Hz, 1H), 7.95 (d, *J* = 6.7 Hz, 1H), 7.88 (t, *J* = 5.6 Hz, 1H), 7.58 (s, 1H), 7.55 (dd, *J* = 8.4, 7.2 Hz, 1H), 7.34 (dd, *J* = 8.2, 1.0 Hz, 1H), 7.06 (d, *J* = 8.6 Hz, 1H), 7.02 (d, *J* = 7.0 Hz, 1H), 6.53 (t, *J* = 5.6 Hz, 1H), 5.02 (dd, *J* = 12.9, 5.4 Hz, 1H), 3.54 (t, *J* = 5.4 Hz, 2H), 3.42 (t, *J* = 5.8 Hz, 2H), 3.39 – 3.36 (m, 2H), 3.23 – 3.19 (m, 2H), 3.04 (t, *J* = 7.5 Hz, 2H), 2.83 (ddd, *J* = 17.4, 14.1, 5.3 Hz, 1H), 2.56 – 2.48 (m, 4H), 2.02 – 1.97 (m, 1H).
**3**	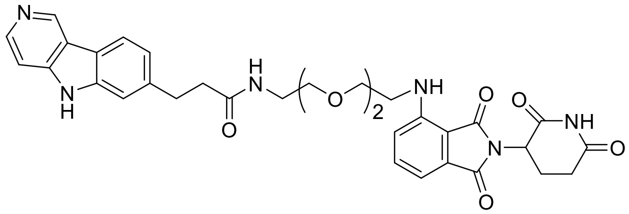<br>**QC-01-175**<br>N-(2-(2-(2-((2-(2,6-dioxopiperidin-3-yl)-1,3-dioxoisoindolin-4-yl)amino)ethoxy)ethoxy)ethyl)-3-(5H-pyrido[4,3-b]indol-7-yl)propanamide	^{1}H NMR (500 MHz, DMSO) δ 13.04 (s, 1H), 11.09 (s, 1H), 9.67 (s, 1H), 8.62 (d, *J* = 6.6 Hz, 1H), 8.31 (d, *J* = 8.1 Hz, 1H), 7.96 (d, *J* = 6.7 Hz, 1H), 7.90 (t, *J* = 5.6 Hz, 1H), 7.58 (s, 1H), 7.56 (dd, *J* = 8.3, 7.4 Hz, 1H), 7.33 (d, *J* = 7.4 Hz, 1H), 7.11 (d, *J* = 8.6 Hz, 1H), 7.01 (d, *J* = 7.0 Hz, 1H), 6.57 (t, *J* = 5.7 Hz, 1H), 5.05 (dd, *J* = 12.7, 5.4 Hz, 1H), 3.58 (t, *J* = 5.7 Hz, 2H), 3.52 – 3.50 (m, 2H), 3.47 – 3.42 (m, 6H), 3.20 – 3.17 (m, 2H), 3.03 (t, *J* = 7.6 Hz, 2H), 2.87 (ddd, *J* = 16.9, 13.8, 5.3 Hz, 1H), 2.60 – 2.55 (m, 1H), 2.54 – 2.47 (m, 3H), 2.01 (ddd, *J* = 10.0, 6.7, 4.0 Hz, 1H).

**4**	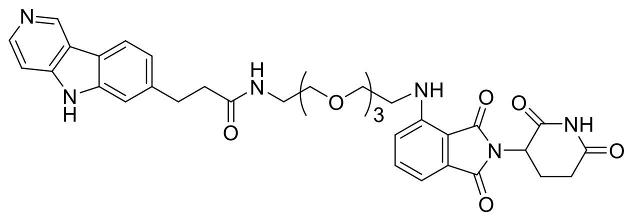 **QC-01-208** N-(2-(2-(2-(2-((2-(2,6-dioxopiperidin-3-yl)-1,3-dioxoisoindolin-4-yl)amino)ethoxy)ethoxy)ethoxy)ethyl)-3-(5H-pyrido[4,3-b]indol-7-yl)propanamide	^{1}H NMR (500 MHz, DMSO) δ 13.02 (s, 1H), 11.09 (s, 1H), 9.67 (s, 1H), 8.62 (d, *J* = 6.7 Hz, 1H), 8.32 (d, *J* = 8.1 Hz, 1H), 7.96 (d, *J* = 6.7 Hz, 1H), 7.91 (t, *J* = 5.6 Hz, 1H), 7.59 (s, 1H), 7.56 (dd, *J* = 8.4, 7.2 Hz, 1H), 7.34 (d, *J* = 9.0 Hz, 1H), 7.11 (d, *J* = 8.6 Hz, 1H), 7.02 (d, *J* = 7.0 Hz, 1H), 6.57 (br s, 1H), 5.05 (dd, *J* = 12.8, 5.4 Hz, 1H), 3.60 (t, *J* = 5.4 Hz, 2H), 3.55 – 3.53 (m, 2H), 3.50 – 3.48 (m, 2H), 3.46 – 3.42 (m, 6H), 3.35 (t, *J* = 5.8 Hz, 2H), 3.19 – 3.16 (m, *J* = 5.7 Hz, 2H), 3.03 (t, *J* = 7.6 Hz, 2H), 2.88 (ddd, *J* = 17.0, 13.9, 5.4 Hz, 1H), 2.60 – 2.56 (m, 1H), 2.54 – 2.47 (m, 3H), 2.06 – 1.96 (m, 1H).
**5**	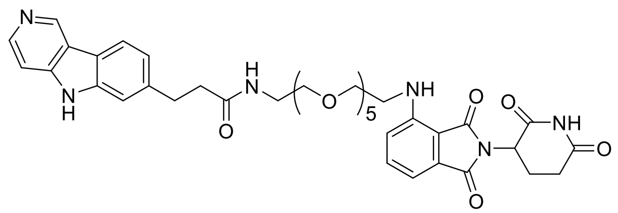 **QC-01-176** N-(17-((2-(2,6-dioxopiperidin-3-yl)-1,3-dioxoisoindolin-4-yl)amino)-3,6,9,12,15-pentaoxaheptadecyl)-3-(5H-pyrido[4,3-b]indol-7-yl)propanamide	^{1}H NMR (500 MHz, DMSO) δ 13.01 (s, 1H), 9.67 (s, 1H), 8.62 (d, *J* = 6.8 Hz, 1H), 8.32 (d, *J* = 8.1 Hz, 1H), 7.96 (d, *J* = 6.7 Hz, 1H), 7.91 (t, *J* = 5.6 Hz, 1H), 7.59 (s, 1H), 7.56 (dd, *J* = 8.5, 7.2 Hz, 1H), 7.35 (dd, *J* = 8.2, 1.1 Hz, 1H), 7.12 (d, *J* = 8.6 Hz, 1H), 7.02 (d, *J* = 6.9 Hz, 1H), 6.58 (t, *J* = 5.7 Hz, 1H), 5.05 (dd, *J* = 12.8, 5.4 Hz, 1H), 3.60 (t, *J* = 5.5 Hz, 2H), 3.56 – 3.54 (m, 2H), 3.52 – 3.37 (m, 16H), 3.35 (t, *J* = 5.9 Hz, 3H), 3.20 – 3.16 (m, 2H), 3.04 (t, *J* = 7.6 Hz, 2H), 2.91 – 2.84 (m, 1H), 2.60 – 2.60 (m, 1H), 2.53 – 2.47 (m, 3H), 2.04 – 2.00 (m, 1H).
**6**	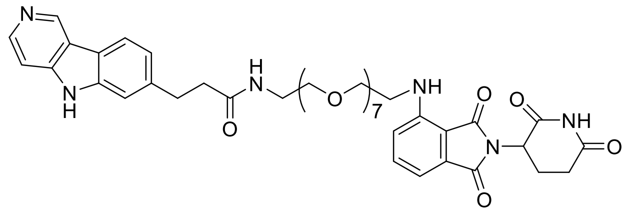 **QC-02-001** N-(23-((2-(2,6-dioxopiperidin-3-yl)-1,3-dioxoisoindolin-4-yl)amino)-3,6,9,12,15,18,21-heptaoxatricosyl)-3-(5H-pyrido[4,3-b]indol-7-yl)propanamide	^{1}H NMR (500 MHz, DMSO) δ 13.03 (s, 1H), 11.09 (s, 1H), 9.67 (s, 1H), 8.62 (d, *J* = 6.8 Hz, 1H), 8.32 (d, *J* = 8.1 Hz, 1H), 7.97 (d, *J* = 6.7 Hz, 1H), 7.92 (t, *J* = 5.6 Hz, 1H), 7.59 (s, 1H), 7.57 (dd, *J* = 9.5, 8.2 Hz, 1H), 7.35 (dd, *J* = 8.2, 1.1 Hz, 1H), 7.13 (d, *J* = 8.6 Hz, 1H), 7.03 (d, *J* = 7.0 Hz, 1H), 6.59 (t, *J* = 5.6 Hz, 1H), 5.05 (dd, *J* = 12.8, 5.4 Hz, 1H), 3.61 (t, *J* = 5.4 Hz, 2H), 3.56 – 3.54 (m, 2H), 3.52 – 3.43 (m, 20H), 3.35 (t, *J* = 5.8 Hz, 2H), 3.20 – 3.16 (m, 1H), 3.04 (t, *J* = 7.6 Hz, 1H), 2.88 (ddd, *J* = 16.9, 13.8, 5.4 Hz, 1H), 2.60 – 2.56 (m, 1H), 2.54 – 2.47 (m, 3H), 2.04 – 1.99 (m, 1H).
**7**	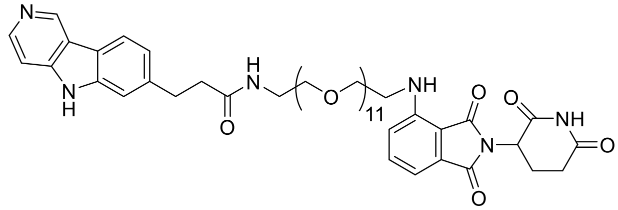	^{1}H NMR (500 MHz, DMSO) δ 13.02 (s, 1H), 11.09 (s, 1H), 9.68 (s, 1H), 8.62 (d, *J* = 6.7 Hz, 1H), 8.32 (d, *J* = 8.1 Hz, 1H), 7.97 (d, *J* = 6.7 Hz, 1H), 7.92 (t, *J* = 5.6 Hz, 1H), 7.59 (s, 1H), 7.57 (dd, *J* = 8.6, 7.3 Hz, 1H), 7.35 (d, *J* = 8.1 Hz, 1H), 7.13 (d, *J* = 8.6 Hz, 1H), 7.03 (d, *J* = 7.0 Hz,

	**QC-02-004**<br>N-(35-((2-(2,6-dioxopiperidin-3-yl)-1,3-dioxoisoindolin-4-yl)amino)-3,6,9,12,15,18,21,24,27,30,33-undecaoxapentatriacontyl)-3-(5H-pyrido[4,3-b]indol-7-yl)propanamide	1H), 6.59 (br s, 1H), 5.05 (dd, *J* = 12.8, 5.4 Hz, 1H), 3.61 (t, *J* = 5.4 Hz, 2H), 3.57 – 3.55 (m, 2H), 3.53 – 3.43 (m, 36H), 3.35 (t, *J* = 5.8 Hz, 2H), 3.18 (q, *J* = 5.7 Hz, 2H), 3.04 (t, *J* = 7.6 Hz, 2H), 2.88 (ddd, *J* = 16.9, 13.8, 5.4 Hz, 1H), 2.60 – 2.56 (m, 1H), 2.52 (dd, *J* = 18.3, 13.8 Hz, 3H), 2.04 – 2.00 (m, 1H).

(2S,4R)-1-[(2S)-3,3-Dimethyl-2-(3-{2-[2-(3-{5H-pyrido[4,3-b]indol-7-yl}propanamido)ethoxy]ethoxy}propanamido)butanoyl]-4-hydroxy-N-[(1S)-1-[4-(4-methyl-1,3-thiazol-5-yl)phenyl]ethyl]pyrrolidine-2-carboxamide (8; FMF-05-129)

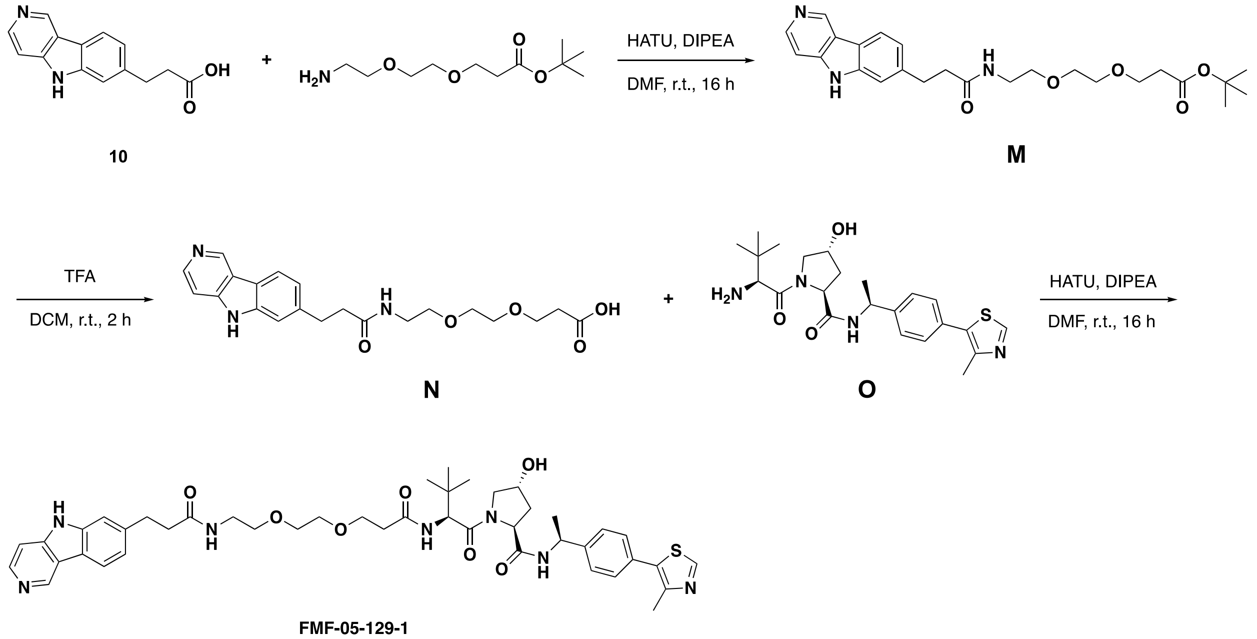

(**M**): To a stirred solution of carboxylic acid **10** (30 mg, 0.125 mmol), HATU (53 mg, 1.38 mmol) and DIPEA (70 μL, 0.375 mmol) in DMF (5 mL) at 25°C was added the corresponding primary amine (33 mg, 0.138 mmol). The reaction mixture was stirred for 16 hrs, diluted with 0.1 N aqueous NaOH (15 mL) and extracted with CH_2Cl_2 (3 x 20 mL). The combined organic phases were dried over anhydrous Na_2SO_4 and concentrated under reduced pressure. The residue was purified by flash column chromatography (0-5 % MeOH in DCM) to give compound **M** (18 mg, 0.040 mmol, 32% yield). MS (ESI) m/z 456 $(M+H)^+$

(**N**): A solution of **M** (18 mg, 0.04 mmol) in CH_2Cl_2 (2 mL) and TFA (1 mL) was stirred at 25°C for 12 hrs before it was concentrated under reduced pressure to give compound **N** (20 mg, 0.04 mmol, quant. yield). MS (ESI) m/z 400 $(M+H)^+$

(2S,4R)-1-[(2S)-3,3-Dimethyl-2-(3-{2-[2-(3-{5H-pyrido[4,3-b]indol-7-yl}propanamido)ethoxy]ethoxy}propanamido)butanoyl]-4-hydroxy-N-[(1S)-1-[4-(4-methyl-1,3-thiazol-5-yl)phenyl]ethyl]pyrrolidine-2-carboxamide (**8**): Compound **O** (25 mg, 0.06 mmol) was added

to a stirred solution of carboxylic acid **N** (20 mg, 0.04 mmol), HATU (27 mg, 0.07 mmol) and DIPEA (35 µL, 0.18 mmol) in DMF (2 mL) at 25°C. The reaction mixture was stirred for 16 hrs, diluted with saturated aqueous $NaHCO_3$ (15 mL) and extracted with CH_2Cl_2 (3 x 20 mL). The combined organic phases were dried over anhydrous Na_2SO_4 and concentrated under reduced pressure. The residue was purified by reverse-phase HPLC to give compound **8** (**FMF-05-129-1**) (10 mg, 0.01 mmol, 25% yield). ^{1}H NMR (500 MHz, DMSO-d_6) δ 9.67 (s, 1H), 8.99 (s, 1H), 8.82 (d, *J* = 6.1 Hz, 1H), 8.37 (dd, *J* = 9.9, 7.9 Hz, 2H), 7.97 (t, *J* = 5.6 Hz, 1H), 7.86 (d, *J* = 9.3 Hz, 1H), 7.75 (d, *J* = 6.1 Hz, 1H), 7.49 (dd, *J* = 8.1, 1.3 Hz, 1H), 7.44 (dd, *J* = 8.2, 1.7 Hz, 3H), 7.40 – 7.37 (m, 3H), 4.91 (h, *J* = 7.0 Hz, 1H), 4.50 (dd, *J* = 26.0, 9.0 Hz, 1H), 4.43 (t, *J* = 8.1 Hz, 1H), 4.36 – 4.25 (m, 2H), 3.66 – 3.56 (m, 3H), 3.54 (s, 6H), 3.51 – 3.35 (m, 6H), 3.21 (q, *J* = 5.8 Hz, 2H), 3.09 (t, *J* = 7.6 Hz, 2H), 2.82 (s, 5H), 2.59 – 2.52 (m, 2H), 2.46 (s, 4H), 2.34 (dt, *J* = 14.8, 6.2 Hz, 1H), 2.19 – 1.97 (m, 1H), 1.76 (dddd, *J* = 38.8, 13.3, 8.9, 4.4 Hz, 1H), 1.38 (d, *J* = 7.0 Hz, 4H), 1.07 (s, 4H), 0.93 (d, *J* = 5.1 Hz, 9H). MS (ESI) m/z 827 $(M+H)^+$

Example compounds **9-14** were prepared in an analogous manner to compound **8**, employing the corresponding amine starting materials and carboxylic acid **J**.

	**Structure/Name**	**Characterization**
**9**	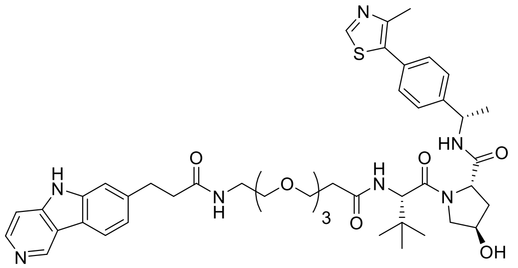<br>**FMF-05-129-2**<br>(2S,4R)-1-[(2S)-3,3-dimethyl-2-[3-(2-{2-[2-(3-{5H-pyrido[4,3-b]indol-7-yl}propanamido)ethoxy]ethoxy}ethoxy)propanamido]butanoyl]-4-hydroxy-N-[(1S)-1-[4-(4-methyl-1,3-thiazol-5-yl)phenyl]ethyl]pyrrolidine-2-carboxamide	^{1}H NMR (500 MHz, DMSO-d_6) δ 13.02 (s, 1H), 9.69 (s, 1H), 8.99 (s, 1H), 8.63 (d, *J* = 6.8 Hz, 1H), 8.36 (dd, *J* = 20.7, 7.9 Hz, 2H), 7.98 (d, *J* = 6.7 Hz, 1H), 7.93 (t, *J* = 5.6 Hz, 1H), 7.86 (d, *J* = 9.3 Hz, 1H), 7.60 (s, 1H), 7.47 – 7.41 (m, 2H), 7.41 – 7.30 (m, 3H), 4.92 (p, *J* = 7.0 Hz, 1H), 4.53 (d, *J* = 9.3 Hz, 1H), 4.43 (t, *J* = 8.1 Hz, 1H), 4.29 (d, *J* = 4.4 Hz, 1H), 3.59 (dddd, *J* = 18.5, 15.9, 7.8, 5.3 Hz, 4H), 3.51 – 3.38 (m, 8H), 3.37 (t, *J* = 5.9 Hz, 2H), 3.25 – 3.14 (m, 2H), 3.05 (t, *J* = 7.6 Hz, 2H), 2.46 (s, 3H), 2.35 (dt, *J* = 14.7, 6.1 Hz, 1H), 2.06 – 1.97 (m, 1H), 1.80 (ddd, *J* = 12.9, 8.5, 4.6 Hz, 1H), 1.37 (d, *J* = 7.0 Hz, 3H), 0.93 (d, *J* = 5.1 Hz, 9H). MS (ESI) m/z 871 $(M+H)^+$
**10**	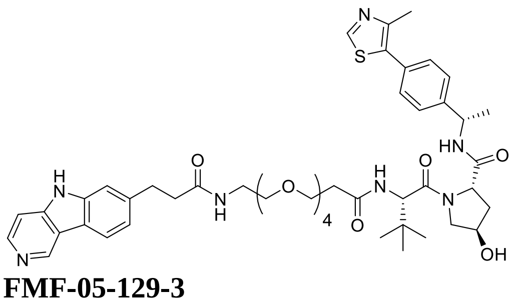<br>**FMF-05-129-3**	^{1}H NMR (500 MHz, DMSO-d_6) δ 13.04 (s, 1H), 9.69 (s, 1H), 8.99 (s, 1H), 8.68 – 8.56 (m, 1H), 8.36 (dd, *J* = 21.4, 7.9 Hz, 2H), 7.98 (d, *J* = 6.8 Hz, 1H), 7.95 – 7.80 (m, 2H), 7.65 – 7.55 (m, 1H), 7.48 – 7.30 (m, 5H), 4.92 (p, *J* = 7.0 Hz, 1H), 4.53 (d, *J* = 9.4 Hz, 1H), 4.43 (t, *J* = 8.1 Hz, 1H), 4.28 (s, 1H), 3.65 – 3.55 (m, 4H), 3.55 – 3.41 (m, 14H), 3.41 – 3.31 (m, 2H), 3.19 (qd, *J* = 5.7, 2.1 Hz, 2H), 3.05 (t, *J* = 7.6

	(2S,4R)-1-[(2S)-3,3-dimethyl-2-[1-(3-{5H-pyrido[4,3-b]indol-7-yl}propanamido)-3,6,9,12-tetraoxapentadecan-15-amido]butanoyl]-4-hydroxy-N-[(1S)-1-[4-(4-methyl-1,3-thiazol-5-yl)phenyl]ethyl]pyrrolidine-2-carboxamide	Hz, 2H), 2.95 (s, 3H), 2.79 (s, 3H), 2.46 (d, *J* = 2.8 Hz, 3H), 2.38 – 2.26 (m, 1H), 2.02 (td, *J* = 9.5, 8.2, 4.5 Hz, 1H), 1.96 (s, 3H), 1.80 (ddd, *J* = 12.9, 8.5, 4.6 Hz, 1H), 1.47 (s, 1H), 1.43 – 1.32 (m, 3H), 0.94 (s, 9H). MS (ESI) m/z 915 $(M+H)^+$
**11**	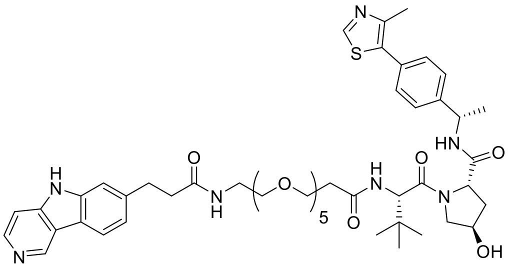 **FMF-05-129-4** (2S,4R)-1-[(2S)-3,3-dimethyl-2-[1-(3-{5H-pyrido[4,3-b]indol-7-yl}propanamido)-3,6,9,12,15,18-hexaoxahenicosan-21-amido]butanoyl]-4-hydroxy-N-[(1S)-1-[4-(4-methyl-1,3-thiazol-5-yl)phenyl]ethyl]pyrrolidine-2-carboxamide	^{1}H NMR (500 MHz, DMSO-d_6) δ 9.80 (s, 1H), 9.00 (d, *J* = 1.3 Hz, 1H), 8.90 (d, *J* = 6.3 Hz, 1H), 8.47 (d, *J* = 7.6 Hz, 1H), 8.39 (dd, *J* = 15.6, 7.9 Hz, 2H), 7.97 (t, *J* = 5.7 Hz, 1H), 7.88 (dd, *J* = 20.7, 7.8 Hz, 2H), 7.53 (dd, *J* = 8.1, 1.3 Hz, 1H), 7.45 – 7.43 (m, 4H), 7.39 – 7.35 (m, 3H), 6.96 (d, *J* = 6.0 Hz, 1H), 4.97 – 4.84 (m, 2H), 4.57 – 4.39 (m, 3H), 4.37 – 4.25 (m, 2H), 4.19 (d, *J* = 6.1 Hz, 1H), 3.86 (d, *J* = 11.4 Hz, 1H), 3.61 (ddt, *J* = 14.1, 11.1, 2.7 Hz, 3H), 3.55 (s, 6H), 3.52 – 3.46 (m, 15H), 3.43 – 3.37 (m, 2H), 3.26 – 3.19 (m, 2H), 3.10 (t, *J* = 7.6 Hz, 2H), 2.83 (s, 6H), 2.59 – 2.52 (m, 2H), 2.46 (s, 5H), 2.35 (dt, *J* = 14.6, 6.1 Hz, 1H), 2.15 (dd, *J* = 13.1, 8.0 Hz, 1H), 2.02 (ddd, *J* = 12.2, 8.2, 2.2 Hz, 1H), 1.76 (dddd, *J* = 38.8, 13.2, 8.9, 4.4 Hz, 1H), 1.38 (dd, *J* = 7.0, 1.7 Hz, 5H), 1.07 (s, 5H), 0.93 (d, *J* = 6.0 Hz, 9H). MS (ESI) m/z 959 $(M+H)^+$
**12**	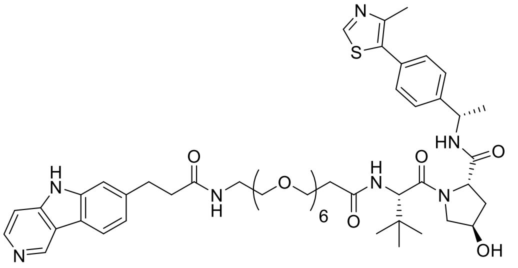 **FMF-05-129-5** (2S,4R)-1-[(2S)-3,3-dimethyl-2-[1-(3-{5H-pyrido[4,3-b]indol-7-yl}propanamido)-3,6,9,12,15,18-hexaoxahenicosan-21-amido]butanoyl]-4-hydroxy-N-[(1S)-1-[4-(4-methyl-1,3-thiazol-5-yl)phenyl]ethyl]pyrrolidine-2-carboxamide	^{1}H NMR (500 MHz, DMSO-d_6) δ 11.11 (s, 1H), 9.81 (s, 1H), 9.00 (d, *J* = 3.3 Hz, 1H), 8.91 – 8.85 (m, 1H), 8.40 (dd, *J* = 14.9, 7.9 Hz, 2H), 8.01 (t, *J* = 5.6 Hz, 1H), 7.88 (dd, *J* = 20.0, 7.8 Hz, 2H), 7.55 – 7.46 (m, 1H), 7.45 – 7.42 (m, 2H), 7.39 (dq, *J* = 8.7, 2.2 Hz, 2H), 4.96 – 4.87 (m, 1H), 4.56 – 4.46 (m, 1H), 4.43 (t, *J* = 8.0 Hz, 1H), 4.30 – 4.23 (m, 1H), 3.55 (s, 5H), 3.52 – 3.42 (m, 14H), 3.38 (t, *J* = 5.8 Hz, 1H), 3.21 (d, *J* = 5.0 Hz, 2H), 3.15 (d, *J* = 20.9 Hz, 2H), 3.10 (t, *J* = 7.6 Hz, 1H), 2.83 (s, 5H), 2.46 (s, 3H), 1.41 – 1.34 (m, 4H), 1.00 (s, 3H), 0.94 (s, 9H). MS (ESI) m/z 1003 $(M+H)^+$

**13**	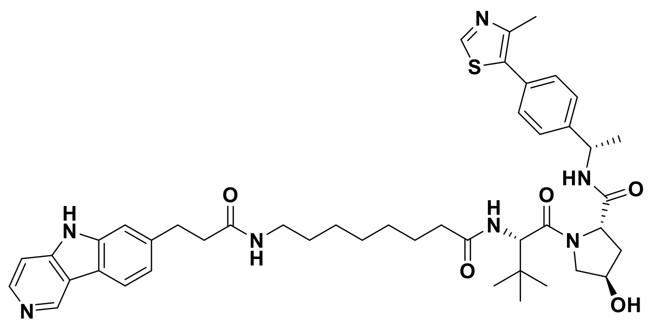<br>**FMF-06-066-1**<br>(2S,4R)-1-((S)-2-(8-(3-(5H-pyrido[4,3-b]indol-7-yl)propanamido)octanamido)-3,3-dimethylbutanoyl)-4-hydroxy-N-((S)-1-(4-(4-methylthiazol-5-yl)phenyl)ethyl)pyrrolidine-2-carboxamide	^{1}H NMR (500 MHz, DMSO-d_6) δ 13.05 (s, 1H), 9.69 (s, 1H), 8.99 (s, 1H), 8.63 (d, *J* = 6.8 Hz, 1H), 8.37 (d, *J* = 7.7 Hz, 1H), 8.33 (d, *J* = 8.1 Hz, 1H), 7.97 (d, *J* = 6.7 Hz, 1H), 7.84 – 7.74 (m, 2H), 7.59 (s, 1H), 7.45 – 7.42 (m, 2H), 7.40 – 7.36 (m, 3H), 4.92 (p, *J* = 7.2 Hz, 1H), 4.51 (d, *J* = 9.3 Hz, 1H), 4.43 (t, *J* = 8.0 Hz, 1H), 4.29 (s, 1H), 4.07 – 3.96 (m, 2H), 3.07 – 2.98 (m, 5H), 2.55 (s, 1H), 2.46 (d, *J* = 2.3 Hz, 4H), 2.25 – 2.16 (m, 1H), 2.10 – 1.95 (m, 3H), 1.80 (ddd, *J* = 12.8, 8.5, 4.6 Hz, 1H), 1.38 (dd, *J* = 6.8, 3.8 Hz, 5H), 1.24 (s, 3H), 1.08 – 0.97 (m, 3H), 0.93 (s, 9H). MS (ESI) m/z 809 $(M+H)^+$
**14**	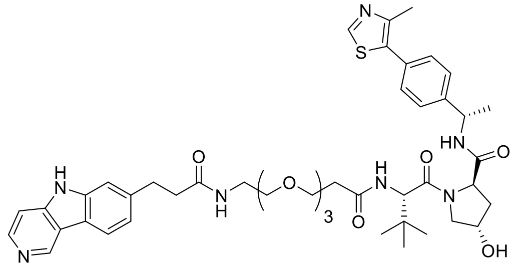<br>**FMF-06-064-1**<br>(2R,4S)-1-((S)-2-(tert-butyl)-4,17-dioxo-19-(5H-pyrido[4,3-b]indol-7-yl)-7,10,13-trioxa-3,16-diazanonadecanoyl)-4-hydroxy-N-((S)-1-(4-(4-methylthiazol-5-yl)phenyl)ethyl)pyrrolidine-2-carboxamide	^{1}H NMR (500 MHz, DMSO-d_6) δ 13.07 (s, 1H), 9.68 (s, 1H), 8.99 (d, *J* = 6.4 Hz, 2H), 8.62 (d, *J* = 6.8 Hz, 1H), 8.33 (d, *J* = 8.2 Hz, 1H), 8.07 (d, *J* = 7.9 Hz, 1H), 7.99 – 7.96 (m, 2H), 7.59 (s, 1H), 7.44 (d, *J* = 3.5 Hz, 4H), 7.38 – 7.33 (m, 1H), 4.92 (dt, *J* = 14.5, 7.1 Hz, 2H), 4.45 – 4.27 (m, 4H), 3.38 – 3.31 (m, 2H), 3.04 (t, *J* = 7.8 Hz, 2H), 2.79 (s, 2H), 2.47 – 2.43 (m, 5H), 2.29 (dt, *J* = 13.4, 6.2 Hz, 2H), 2.11 – 1.86 (m, 4H), 1.31 (d, *J* = 7.2 Hz, 3H), 1.24 (s, 3H), 1.10 (t, *J* = 7.0 Hz, 1H), 1.00 (d, *J* = 2.1 Hz, 5H), 0.96 (d, *J* = 3.1 Hz, 9H). MS (ESI) m/z 871 $(M+H)^+$

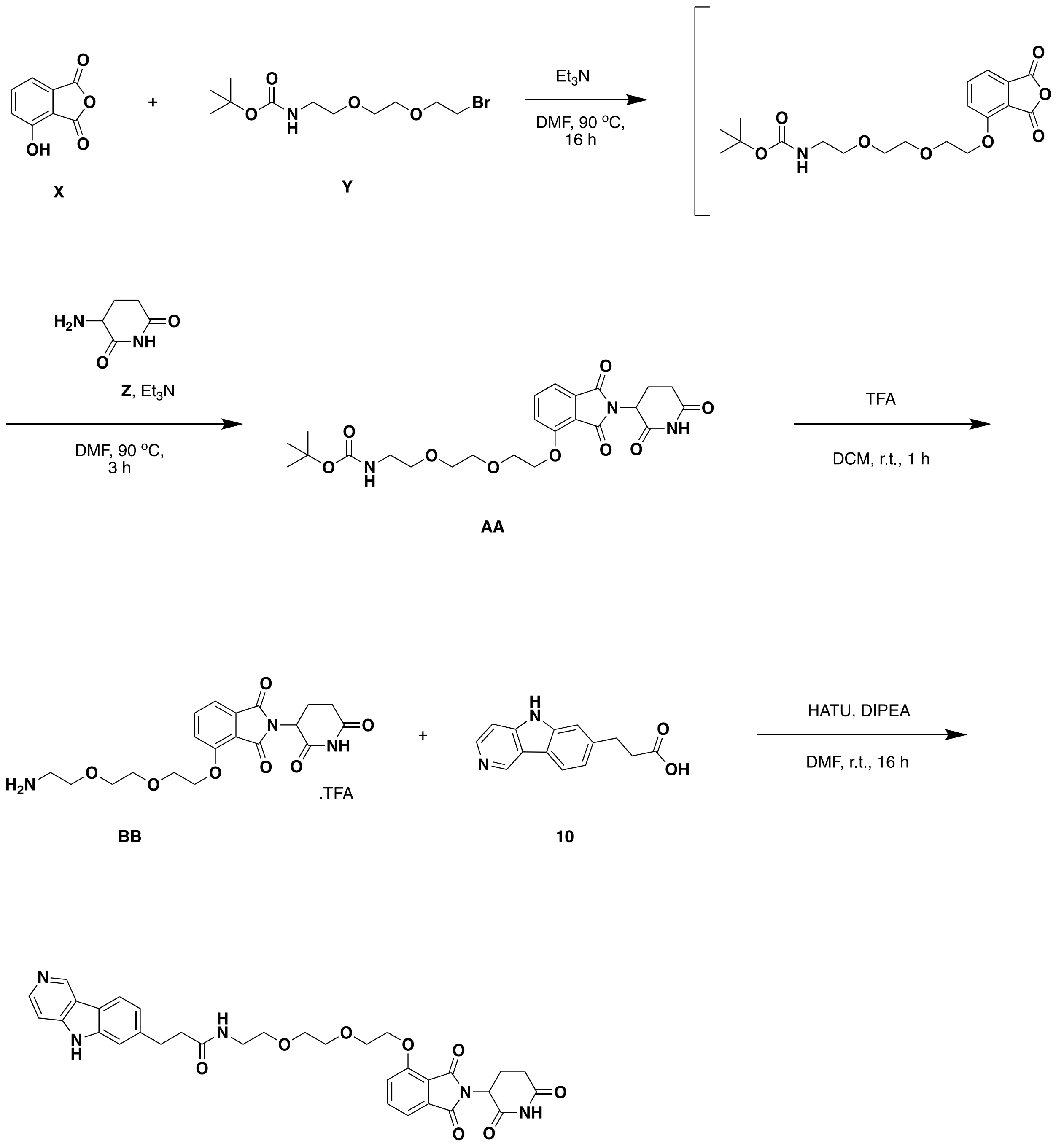

FMF-06-038-1

(**AA**): Compound **X** (100 mg, 0.61mmol), compound **Y** (256 mg, 0.92 mmol), and Et_3N (430μL, 3.06 mmol) were stirred in DMF (2 mL) at 90°C for 16 hrs. The reaction mixture was cooled to room temperature and filtered. The precipitate was washed with EtOAc (20 mL) and the combined filtrate and organics were concentrated under reduced pressure to 2 mL volume. Et_3N (430 μL, 3.06 mmol) and compound **Z** (250 mg, 0.53 mmol) were added to the concentrated filtrates and the reaction mixture heated at 90°C for 3 hrs. The reaction was concentrated under reduced pressure and purified by flash chromatography to afford the

title compound (270 mg, 0.57 mmol, 93%). ^{1}H NMR (500 MHz, DMSO-d_6) δ 11.10 (s, 1H), 7.82 (dd, *J* = 8.5, 7.2 Hz, 1H), 7.56 – 7.51 (m, 1H), 7.46 (d, *J* = 7.2 Hz, 1H), 6.77 – 6.70 (m, 1H), 5.09 (dd, *J* = 12.8, 5.5 Hz, 1H), 4.37 – 4.33 (m, 2H), 3.84 – 3.79 (m, 2H), 3.64 (dd, *J* = 5.8, 3.8 Hz, 2H), 3.54 – 3.48 (m, 4H), 3.37 (d, *J* = 6.2 Hz, 2H), 3.06 (q, *J* = 5.8 Hz, 3H), 2.60 (ddd, *J* = 17.0, 4.4, 2.4 Hz, 1H), 2.03 (dtd, *J* = 13.0, 5.3, 2.2 Hz, 1H), 1.36 (s, 9H). MS (ESI) m/z 506 $(M+H)^+$

(**BB**): Compound **AA** (156 mg, 0.31 mmol) was dissolved in 2 mL DCM and 2 mL TFA, and stirred at room temperature for 2 hrs. The reaction mixture was concentrated under reduced pressure to afford the compound **BB** (161mg, 0.31 mmol, 100%), which was used without further purification. MS (ESI) m/z 406 $(M+H)^+$

N-{2-[2-(2-{[2-(2,6-dioxopiperidin-3-yl)-1,3-dioxo-2,3-dihydro-1H-isoindol-4-yl]oxy}ethoxy)ethoxy]ethyl}-3-{5H-pyrido[4,3-b]indol-7-yl}propanamide (FMF-06-038-1):

Compound **BB** (10 mg, 0.042 mmol), Compound **J** (24 mg, 0.046 mmol), HATU (20 mg, 0.05 mmol), and DIPEA (26 µL, 0.126 mmol) were dissolved in DMF (2 mL) and stirred at room temperature for 4 hrs. The reaction mixture was filtered and purified by HPLC to afford compound **20** (**FMF-06-038-1**) as a TFA salt. (3 mg, 0.004 mmol, 10%). ^{1}H NMR (500 MHz, DMSO-d_6) δ 13.05 (s, 1H), 11.10 (s, 1H), 9.68 (s, 1H), 8.62 (d, *J* = 6.7 Hz, 1H), 8.32 (d, *J* = 8.1 Hz, 1H), 7.97 (d, *J* = 6.7 Hz, 1H), 7.93 (q, *J* = 5.6 Hz, 1H), 7.81 – 7.77 (m, 1H), 7.59 (d, *J* = 4.2 Hz, 1H), 7.51 (d, *J* = 8.6 Hz, 1H), 7.45 – 7.41 (m, 1H), 7.37 – 7.32 (m, 1H), 5.08 (dd, *J* = 12.8, 5.3 Hz, 1H), 4.33 – 4.27 (m, 2H), 3.64 – 3.59 (m, 6H), 3.17 – 3.13 (m, 6H), 3.07 – 3.02 (m, 2H), 2.90 – 2.84 (m, 2H), 2.61 – 2.57 (m, 1H), 2.06 – 2.01 (m, 1H). MS (ESI) m/z 626 $(M-H)^-$

Example compounds **21-24** were prepared in an analogous manner to compound **20**, employing the corresponding amine starting materials and carboxylic acid **J**.

	**Structure/Name**	**Characterization**
**21**	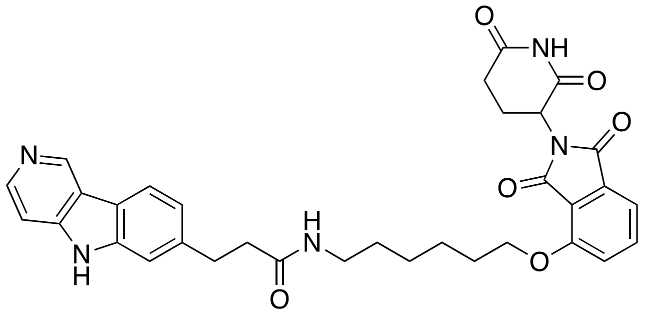 **FMF-06-049-1** N-(6-{[2-(2,6-dioxopiperidin-3-yl)-1,3-dioxo-2,3-dihydro-1H-isoindol-4-yl]oxy}hexyl)-3-{5H-pyrido[4,3-b]indol-7-yl}propanamide	^{1}H NMR (500 MHz, DMSO-d_6) δ 13.04 (s, 1H), 11.10 (s, 1H), 9.67 (s, 1H), 8.61 (d, *J* = 6.7 Hz, 1H), 8.33 (d, *J* = 8.0 Hz, 1H), 7.97 (d, *J* = 6.6 Hz, 1H), 7.81 (dd, *J* = 8.4, 7.2 Hz, 1H), 7.70 (s, 1H), 7.60 (s, 1H), 7.46 (dd, *J* = 13.7, 7.9 Hz, 2H), 7.36 (dd, *J* = 8.3, 1.3 Hz, 1H), 5.08 (dd, *J* = 12.8, 5.5 Hz, 1H), 4.23 – 3.99 (m, 3H), 3.03 (dq, *J* = 12.4, 6.6, 5.9 Hz, 4H), 2.77 (d, *J* = 12.2 Hz, 2H), 1.64 (t, *J* = 7.5 Hz, 1H), 1.53 (d, *J* = 7.2 Hz, 1H), 1.46 (dd, *J* = 15.6, 6.6 Hz, 1H), 1.37 – 1.33 (m, 5H), 1.23 (d, *J* = 11.1 Hz, 2H), 1.11 – 1.04 (m, 3H). MS (ESI) m/z 596 $(M+H)^+$

**22**	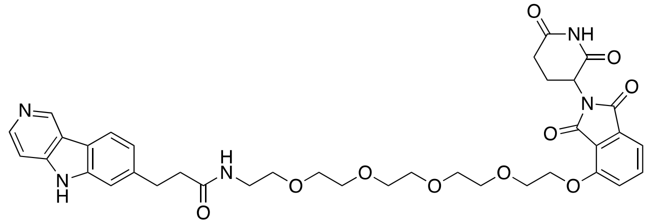 **FMF-06-050-2** N-(2-{2-[2-(2-{[2-(2,6-dioxopiperidin-3-yl)-1,3-dioxo-2,3-dihydro-1H-isoindol-4-yl]oxy}ethoxy)ethoxy]ethoxy}ethyl)-3-{5H-pyrido[4,3-b]indol-7-yl}propanamide	MS (ESI) m/z 672 (M+H)+
**23**	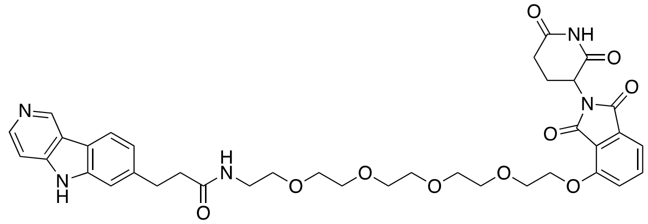 **FMF-06-052-1** N-(14-{[2-(2,6-dioxopiperidin-3-yl)-1,3-dioxo-2,3-dihydro-1H-isoindol-4-yl]oxy}-3,6,9,12-tetraoxatetradecan-1-yl)-3-{5H-pyrido[4,3-b]indol-7-yl}propanamide	^{1}H NMR (500 MHz, DMSO-d_6) δ 12.98 (s, 1H), 11.10 (s, 1H), 9.66 (s, 1H), 8.61 (s, 1H), 8.32 (dd, J = 11.7, 8.1 Hz, 1H), 7.92 (t, J = 5.7 Hz, 1H), 7.80 (dd, J = 8.5, 7.3 Hz, 1H), 7.58 (s, 1H), 7.51 (d, J = 8.5 Hz, 1H), 7.45 (d, J = 7.3 Hz, 1H), 7.33 (dd, J = 8.1, 1.4 Hz, 1H), 5.08 (dd, J = 12.8, 5.4 Hz, 1H), 4.33 (t, J = 4.7 Hz, 2H), 3.79 (t, J = 4.6 Hz, 2H), 3.63 (dd, J = 5.8, 3.7 Hz, 2H), 3.58 – 3.41 (m, 13H), 3.18 (q, J = 5.7 Hz, 2H), 3.04 (t, J = 7.7 Hz, 2H), 2.06 – 1.99 (m, 0H), 1.26 – 1.23 (m, 2H), 1.18 – 0.82 (m, 2H). MS (ESI) m/z 716 (M+H)+
**24**	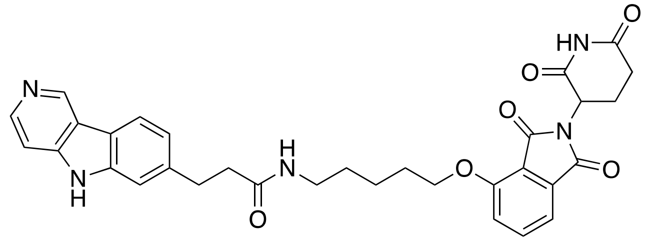 **FMF-06-053-1** N-(5-{[2-(2,6-dioxopiperidin-3-yl)-1,3-dioxo-2,3-dihydro-1H-isoindol-4-yl]oxy}pentyl)-3-{5H-pyrido[4,3-b]indol-7-yl}propanamide	^{1}H NMR (500 MHz, DMSO-d_6) δ 13.12 (s, 1H), 11.10 (s, 1H), 9.66 (s, 1H), 8.60 (d, J = 6.8 Hz, 1H), 8.33 (d, J = 8.1 Hz, 1H), 7.95 (d, J = 6.9 Hz, 1H), 7.85 (t, J = 5.7 Hz, 1H), 7.79 (dd, J = 8.4, 7.3 Hz, 1H), 7.59 (s, 1H), 7.43 (dd, J = 7.9, 4.3 Hz, 2H), 7.35 (dd, J = 8.2, 1.4 Hz, 1H), 5.06 (dd, J = 12.7, 5.4 Hz, 1H), 4.07 (t, J = 6.4 Hz, 2H), 3.08 – 3.01 (m, 4H), 2.92 – 2.85 (m, 4H), 1.67 (p, J = 6.6 Hz, 2H), 1.41 (p, J = 6.9 Hz, 2H), 1.33 (p, J = 7.6 Hz, 1H), 1.29 – 1.21 (m, 4H). MS (ESI) m/z 582 (M+H)+
