# Supplementary material for: Discovery and Optimization of Tau Targeted Protein Degraders Enabled by Patient Induced Pluripotent Stem Cells-Derived Neuronal Models of Tauopathy
Source: Front Cell Neurosci. 2022 Mar 3;16:801179. doi: 10.3389/fncel.2022.801179 (PMC8934437; doi:10.3389/fncel.2022.801179)
Supplement: Supplementary file 2 [file Data_Sheet_2.docx]

**Supplementary Figures**

**
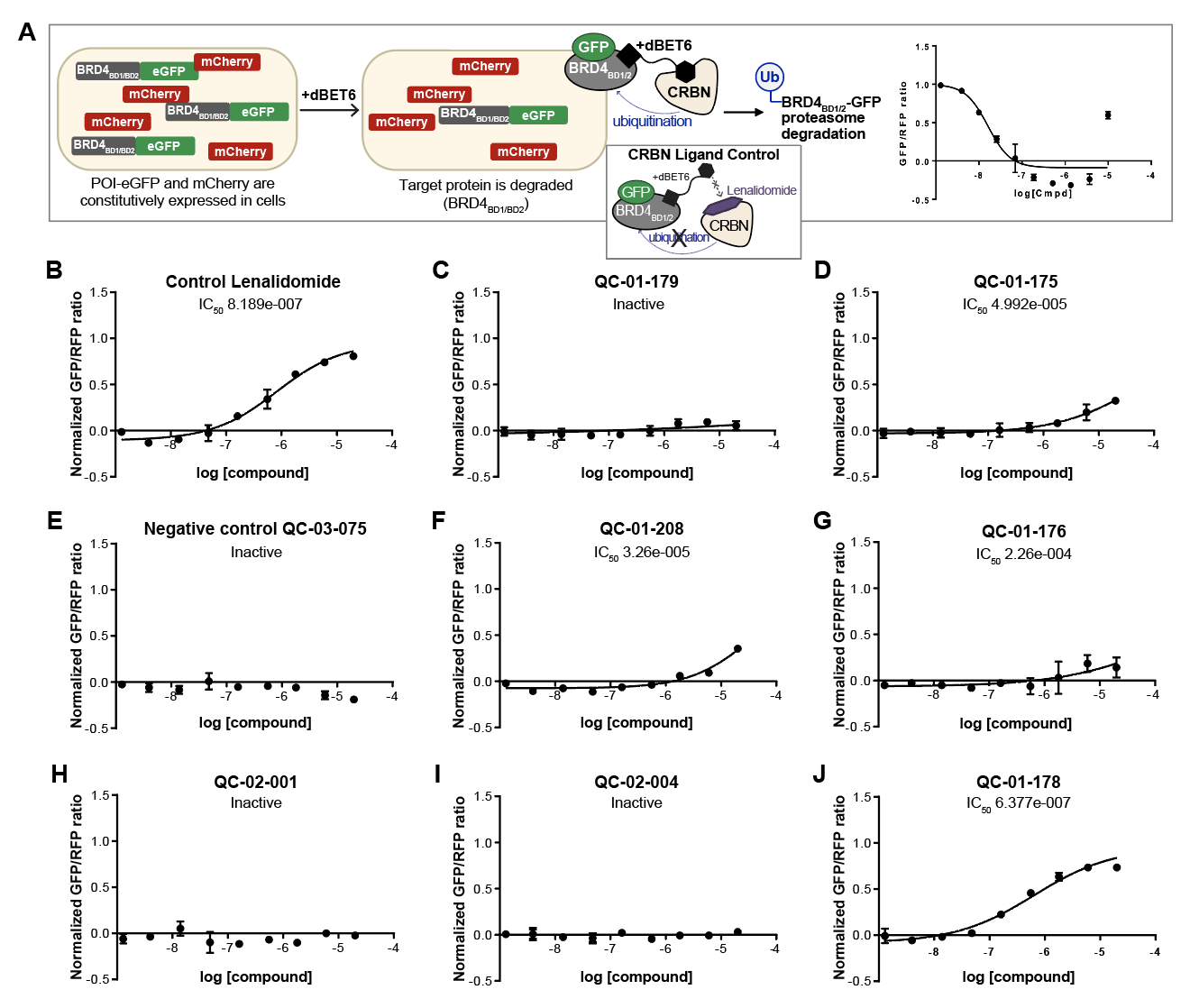
**

**Supplementary Figure 1.** Cellular permeability assay for CRBN-recruiting degraders. **(A)** Schematic of the in vitro assay implemented to evaluate cellular permeability and CRBN engagement for each degrader molecule. **(B-J)** Intracellular ligase engagement assay. BRD4^BD2^-GFP reporter cells were treated with increasing concentrations of lenalidomide or the indicated compound for 5 hrs in the presence of dBET6 (CRBN). Relative abundance of BRD4^BD2^-GFP was measured by FACS. Data are represented as mean GFP/RFP ± SD of three replicates (*n* = 3).

*
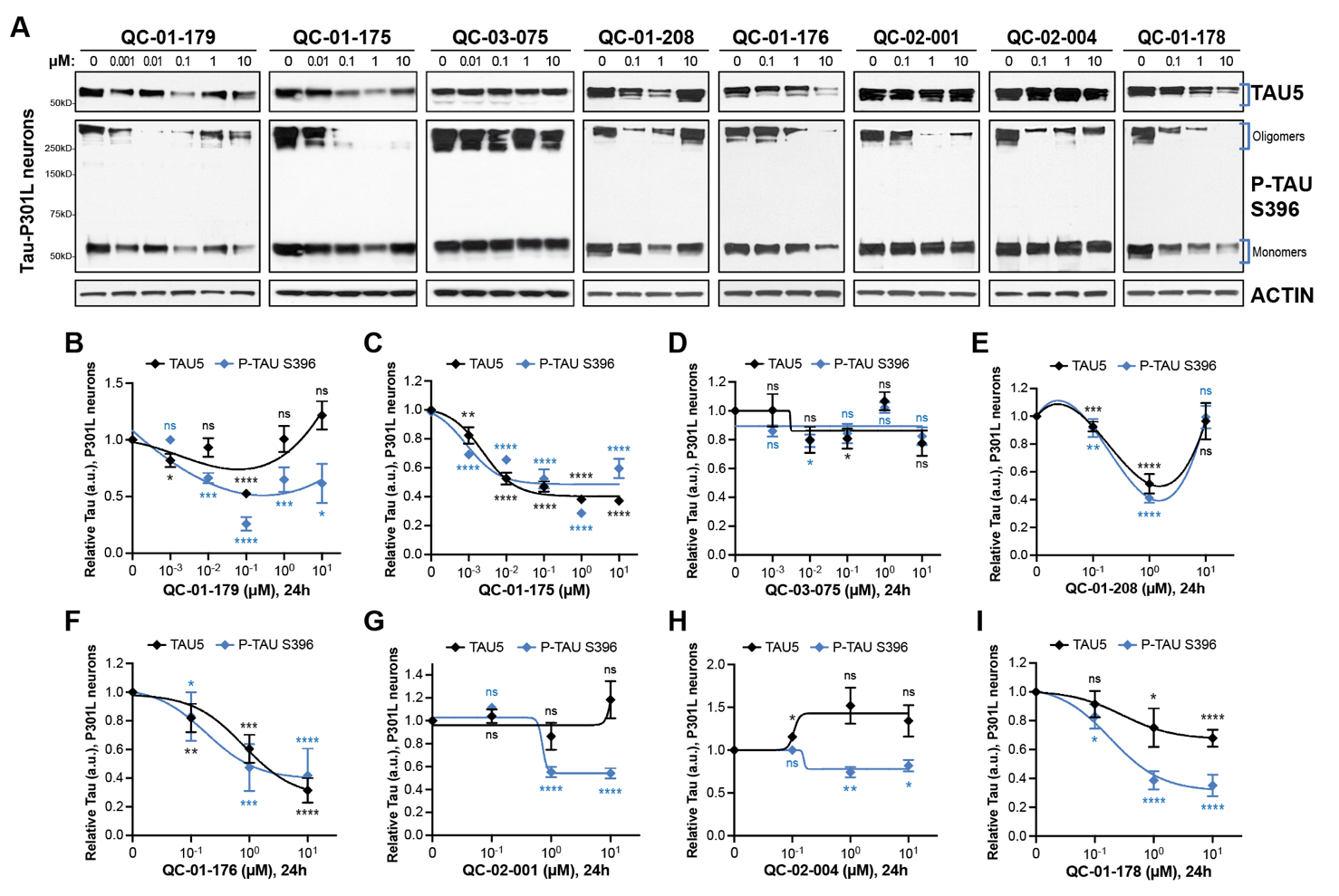
*

**Supplementary Figure 2.** Concentration effect of the QC-Series degraders on tau protein levels of P301L neurons (6-week differentiated). Analysis of total tau (TAU5) and P-tau S396 levels upon treatment for 24 hrs. Representative western blots are shown (**A**) and brackets indicate bands corresponding to quantified TAU5 and P-tau S396 densitometry (**B-I**). Data points represent mean densitometry normalized to actin and relative to vehicle ± SD (*n* ≥ 2). All 0 μM degrader data points correspond to vehicle alone (DMSO) control treatment. Statistics: 2-tailed unpaired Student *t*-test for each concentration relative to vehicle; ^ns^*P*> 0.05, **P*<0.05, ***P*<0.01, ****P*<0.001, *****P*<0.0001.

**Supplementary Figure 3.** Second-generation CRBN degraders cellular permeability and concentration effect in FTD neurons. **(A-F)** Intracellular ligase engagement assay. BRD4^BD2^-GFP reporter cells were treated with increasing concentrations of lenalidomide or the indicated compound for 5 hrs in the presence of dBET6 (CRBN). Relative abundance of BRD4^BD2^-GFP was measured by FACS. Data are represented as mean GFP/RFP ± SD of three replicates (*n* = 3). **(G)** Degraders’ concentration effect on viability of tau-P301L neurons (6-week differentiated) treated for 24 hrs. **(H-M)** Concentration effect of the FMF-06-series degraders on tau protein levels of P301L neurons (6-week differentiated). Analysis of total tau (TAU5) and P-tau S396 levels upon treatment for 24 hrs. Representative western blots are shown (**H**) and brackets indicate bands corresponding to quantified TAU5 and P-tau S396 densitometry (**I-M**). Data points represent mean densitometry normalized to actin and relative to vehicle ± SD (*n* ≥ 2). All 0 μM degrader data points correspond to vehicle alone (DMSO) control treatment. Statistics: 2-tailed unpaired Student *t*-test for each concentration relative to vehicle; ^ns^*P*> 0.05, **P*<0.05, ***P*<0.01, ****P*<0.001, *****P*<0.0001.


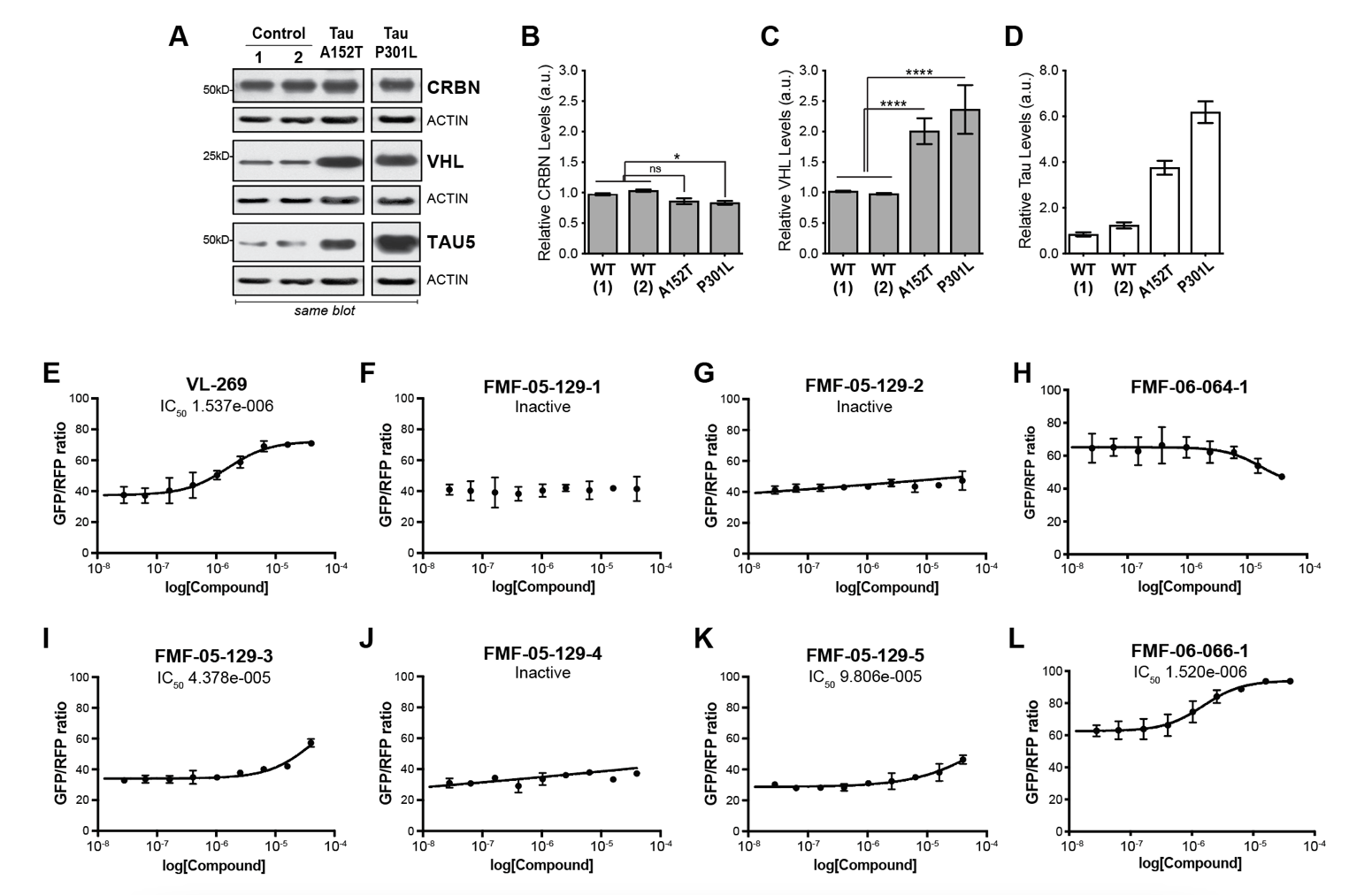


**Supplementary Figure 4.** Expression of E3-ubiquitin ligases VHL and CRBN in control and FTD iPSC-derived neurons, and VHL-targeting degraders cellular permeability. **(A-D)** Western blot and densitometry analysis of CRBN and VHL protein levels in tau-WT (control-1 and -2 lines), tau-A152T and tau-P301L iPSC-derived neurons at 5 weeks of differentiation, and comparative analysis with genotype-dependent tau accumulation (**D**). Graph bars represent mean densitometry (normalized to actin) and relative to control-1 ± SD (*n* = 3). Statistics: 2-tailed unpaired Student *t*-test for each concentration relative to vehicle; ^ns^*P*> 0.05, **P*<0.05, *****P*<0.0001. **(E-L)** Intracellular ligase engagement assay. BRD4^BD2^-GFP reporter cells were treated with increasing concentrations of the indicated compound for 5 hrs in the presence of AT1 (VHL). Relative abundance of BRD4^BD2^-GFP was measured by FACS. Data are represented as mean GFP/RFP ± SD of three replicates (*n* = 3).

**
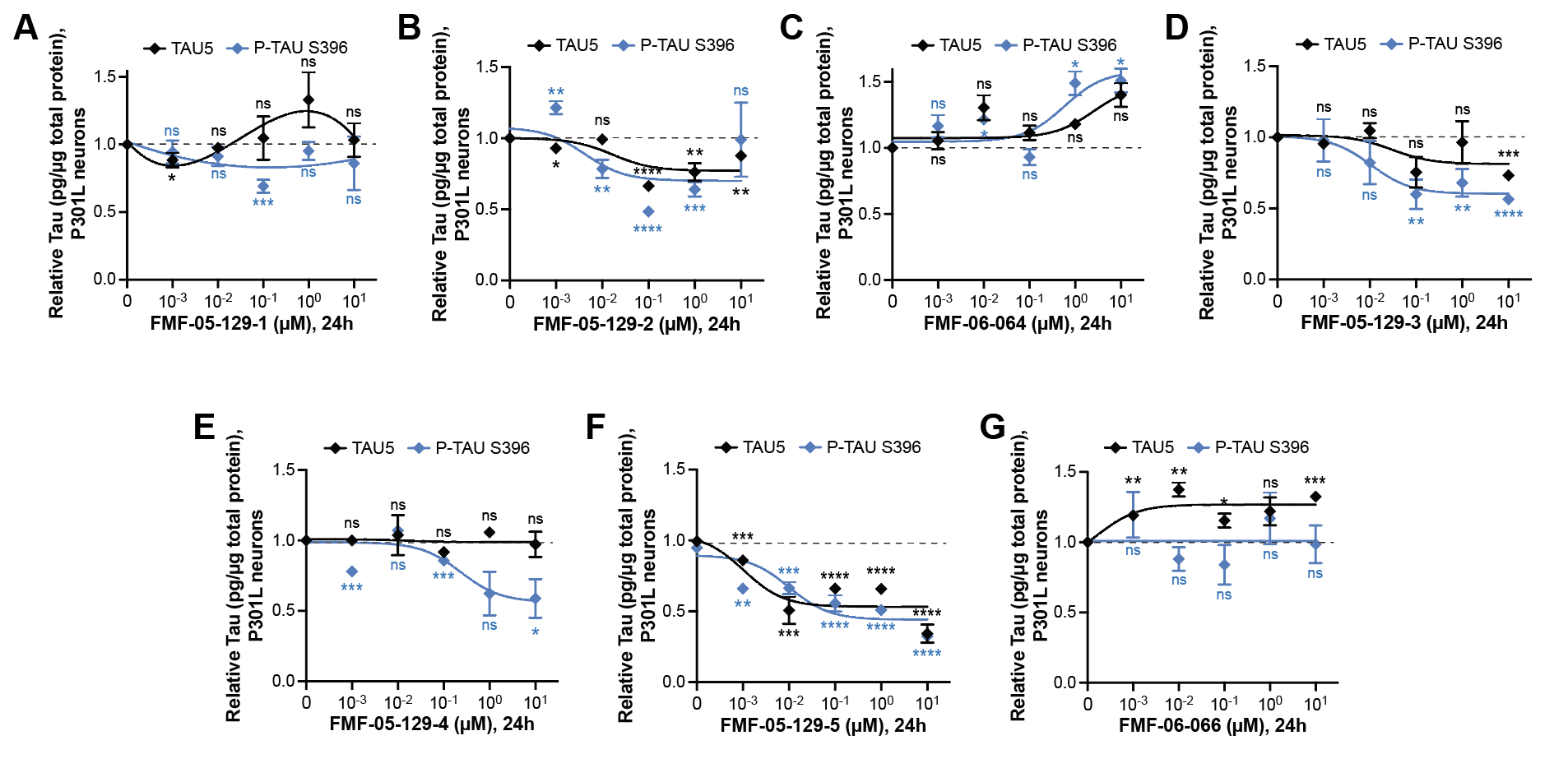
**

**Supplementary Figure 5.** ELISA analysis of concentration effect of VHL-recruiting molecules on total tau (TAU5) and P-tau S396 levels of P301L neurons (6-week differentiated) after 24 hrs of treatment. Data points represent mean tau levels (μg of tau normalized to total protein in the lysate) relative to vehicle samples ± SEM (*n* =3). All 0 μM degrader data points correspond to vehicle alone (DMSO) control treatment. Statistics: 2-tailed unpaired Student t-test for each concentration relative to vehicle; ^ns^*P*> 0.05, **P*<0.05, ***P*<0.01, ****P*<0.001, *****P*<0.0001.
